# The DnaK/DnaJ Chaperone System Enables RNA Polymerase-DksA Complex Formation in Salmonella Experiencing Oxidative Stress

**DOI:** 10.1128/mBio.03443-20

**Published:** 2021-05-11

**Authors:** Ju-Sim Kim, Lin Liu, Andrés Vázquez-Torres

**Affiliations:** a University of Colorado School of Medicine, Department of Immunology & Microbiology, Aurora, Colorado, USA; b Veterans Affairs Eastern Colorado Health Care System, Denver, Colorado, USA; University of Illinois at Chicago

**Keywords:** *Salmonella* Typhimurium, DksA, DnaK, DnaJ, chaperone, stringent response, oxidative stress, redox, hydrogen peroxide

## Abstract

Our previous biochemical approaches showed that the oxidoreductase activity of the DnaJ protein facilitates the interaction of oxidized DksA with RNA polymerase. Investigations herein demonstrate that under biologically relevant conditions the DnaJ- and DksA-codependent activation of the stringent response in Salmonella undergoing oxidative stress involves the DnaK chaperone. Oxidation of DksA cysteine residues stimulates redox-based and holdase interactions with zinc-binding and C-terminal domains of DnaJ. Genetic and biochemical evidence indicates that His^33^ in the HPD motif in the J domain of DnaJ facilitates interactions of unfolded DksA with DnaK. A mutation in His^33^ in the J domain prevents the presentation of unfolded DksA to DnaK without limiting the oxidoreductase activity mapped to DnaJ’s zinc-2 site. Thr^199^ in the ATPase catalytic site of DnaK is required for the formation of the DksA/RNA polymerase complex. The DnaK/DnaJ/DksA complex enables the formation of an enzymatically active RNA polymerase holoenzyme that stimulates transcription of branched-chain amino acid and histidine metabolic genes in Salmonella exposed to reactive oxygen species. The DnaK/DnaJ chaperone protects Salmonella against the cytotoxicity associated with reactive oxygen species generated by the phagocyte NADPH oxidase in the innate host response. The antioxidant defenses associated with DnaK/DnaJ can in part be ascribed to the elicitation of the DksA-dependent stringent response and the protection this chaperone system provides against protein carbonylation in Salmonella undergoing oxidative stress.

## INTRODUCTION

The Hsp70 DnaK is a major chaperone. DnaK together with DnaJ contributes to protein quality control by facilitating folding of nascent proteins, polypeptides emerging through the Sec system, partially unfolded proteins, and protein aggregates (1). The DnaK-DnaJ chaperone system also catalyzes the formation and disassembly of protein complexes (1, 2). Binding and release of protein clients by DnaK are regulated by the ATP hydrolytic activity inherent to the nucleotide-binding domain (NBD), which itself is under the allosteric control exerted by the cochaperone DnaJ and the nucleotide exchange factor GrpE (1). In the ATP-bound, open-conformation state, DnaK’s β-sandwich substrate binding domain (SBD) binds to client proteins with low affinity. Tight substrate binding and ATPase activity of DnaK are triggered following the translocation of the α-helical lid from a location proximal to the N-terminal β-sandwich to the C terminus (1, 3, 4). This massive reorganization of DnaK is set in motion after the nucleotide-binding domain and linker regions of DnaK interact with the HPD motif in the J domain of DnaJ (1). Binding of DnaJ to DnaK is further assisted by the J and G/F (Gly/Phe-rich) domains of the former (5). Type I DnaJ molecules also harbor a central cysteine-rich region with four CXXCXGXG (C, cysteine; G, glycine; X, any amino acid) repeats arranged in an antiparallel β-sheet conformation that coordinates two zinc cations (6, 7). The zinc-binding domain of DnaJ binds unfolded substrates, possesses holding activity, and cooperates with DnaK to refold denatured clients (5, 8, 9). In addition, cysteine residues holding the structural zinc-binding domain of type I DnaJ proteins harbor oxidoreductase activity (10). The oxidoreductase activity of DnaJ proteins can be coupled to DnaK function (11) or operate as an independent thiol-disulfide exchange system analogous to thioredoxin-like proteins as described for the J-domain-deficient ZnJ2 protein from chloroplasts (12).

Escherichia coli strains deficient for *dnaK*, *dnaJ*, or *grpE* genes are temperature sensitive, are prone to filamentation, and fail to form a replicative complex for bacteriophage λ (13–16). In a screen for genes that reversed the temperature sensitivity of a Δ*dnaKJ* mutant E. coli strain, Kang and Craig discovered a *dnaK* suppressor gene they named *dksA* (13). The mechanism by which the overexpression of DksA overcomes the heat shock sensitivity and filamentation of Δ*dnaKJ* E. coli has been a mystery for 30 years.

DksA regulates the stringent response in Gram-negative bacteria, a genetic program that aids the adaptation to nutritional starvation, heat shock, and oxidative and nitrosative stress (17–20). The stringent response is characterized by the transcriptional repression of genes encoding translational machinery and the concomitant activation of genes involved in amino acid biosynthesis, virulence programs, and antibiotic resistance (17–20). The stringent response is further regulated through the allosteric interaction of RNA polymerase with guanosine pentaphosphate or tetraphosphate [(p)ppGpp], which in E. coli and Salmonella is synthetized by the enzymes RelA and SpoT. The nucleotide alarmone (p)ppGpp binds to RNA polymerase at a site formed between the interface of the globular and coiled-coil domains of DksA and the β-subunit of RNA polymerase (21, 22). The stringent response in Gram-negative bacteria regulates the kinetics of the DNA open complex through the collision of two aspartic acids at the tip of the coiled-coil domain of DksA with the bridge-helix of RNA polymerase (22).

The DksA protein has multiple functions in the cell, including the regulation of the stringent response, transcriptional fidelity, prevention of conflicts between RNA and DNA polymerases, and DNA strand break repair (23–27). The interaction of DksA with the secondary channel of RNA polymerase promotes antioxidant defenses of Salmonella (28). A biochemical reconstitution system demonstrated that binding of oxidized DksA to RNA polymerase can be facilitated by the oxidoreductase activity of DnaJ itself (27), consistent with the idea that the foldase and oxidoreductase activities mapped to the zinc-binding domain (9, 12) may be sufficient to promote DksA-RNA polymerase complexes. In the investigations described below, we tested whether DnaJ-dependent activation of RNA polymerase by oxidized DksA is sufficient to stimulate a stringent response in Salmonella undergoing oxidative stress. Our investigations demonstrate that *in vivo*, the chaperone DnaK is required for the DnaJ-DksA redox-based regulation of the stringent response that is elicited upon exposure of Salmonella to reactive oxygen species.

## RESULTS

### DnaK and DnaJ contribute to Salmonella pathogenesis.

**FIG 1:**
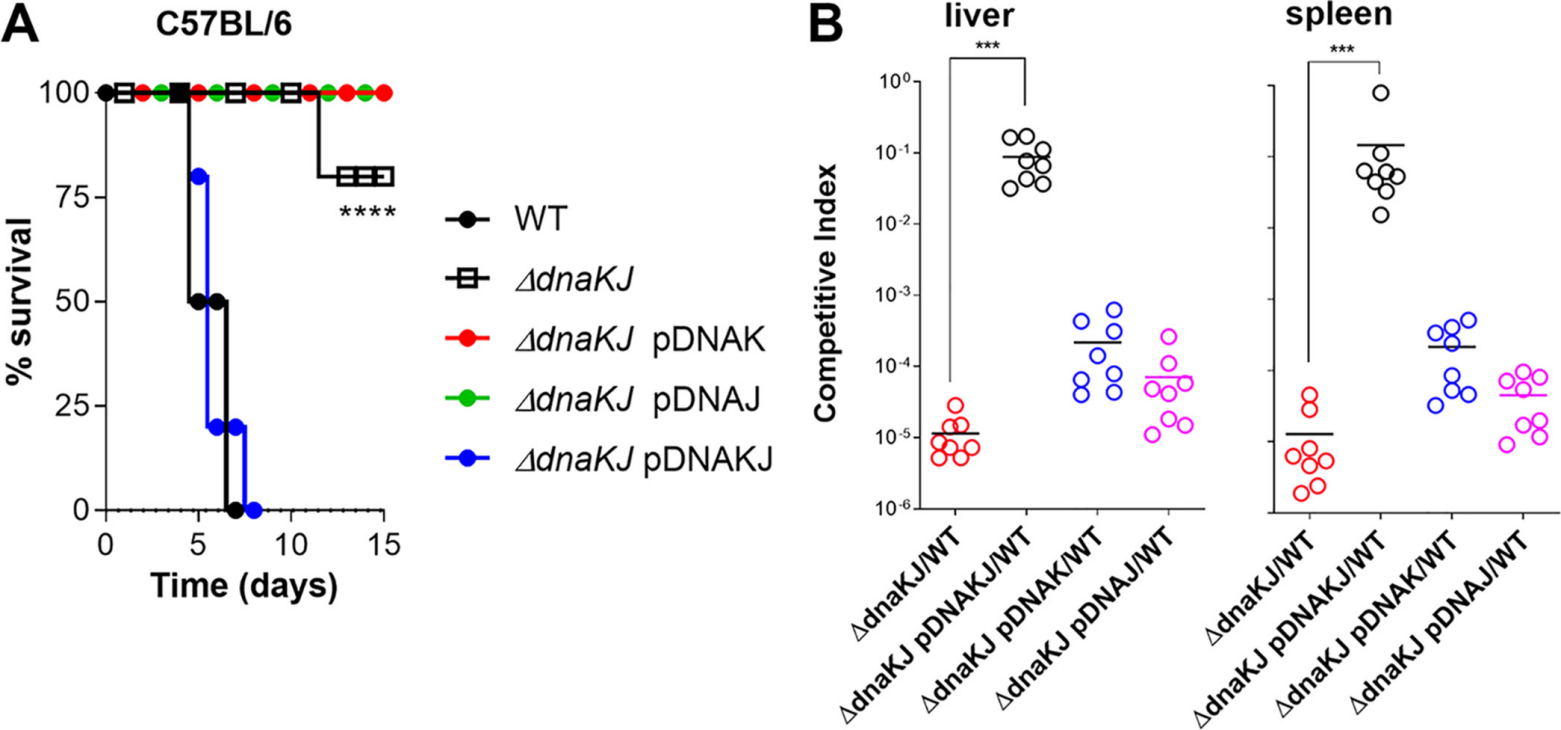
DnaK and DnaJ contribute to Salmonella pathogenesis. (A) Survival of C57BL/6 mice after intraperitoneal (i.p.) inoculation of ∼150 CFU of the indicated Salmonella strains. Mouse survival was monitored over 2 weeks. The data are from 8 to 10 mice from 2 independent experiments. *P* values were determined by log-rank analysis (****, *P < *0.0001) compared to controls infected with wild-type Salmonella. (B) The competitive index was measured in livers and spleens of C57BL/6 mice 96 h after i.p. inoculation of ∼1,000 CFU of a mixture containing equal numbers of the indicated strains. Horizontal bars represent the medians from 8 mice collected in 2 independent experiments. ***, *P < *0.001, as determined by one-way ANOVA.

DnaJ is critical to Salmonella pathogenesis (27). Because DnaJ can work independently or in tandem with DnaK, we studied whether the contribution of DnaJ to Salmonella pathogenesis involves DnaK. To ascertain the roles of *dnaK* and *dnaJ* in Salmonella virulence, we took advantage of the complementation of a Δ*dnaK* Δ*dnaJ* (i.e., Δ*dnaKJ*) double mutant with plasmids expressing *dnaK* and/or *dnaJ* genes (see Fig. S1A and B in the supplemental material). We found that a Δ*dnaKJ* Salmonella strain is attenuated in immunocompetent C57BL/6 mice (Fig. 1A). Strains lacking either *dnaK* or *dnaJ* alleles remained attenuated in C57BL/6 mice. However, Δ*dnaKJ* Salmonella regained virulence upon in *trans* expression of both *dnaK* and *dnaJ* genes from the low-copy-number pWSK29 plasmid (Fig. 1A and Fig. S1A). These findings suggest that both DnaK and DnaJ are required for full Salmonella virulence in mice.

10.1128/mBio.03443-20.5 FIG S1 Determination of DnaK and DnaJ expression of Salmonella strains. Download FIG S1, TIF file, 1.0 MB. Copyright © 2021 Kim et al. 2021 Kim et al. https://creativecommons.org/licenses/by/4.0/ This content is distributed under the terms of the Creative Commons Attribution 4.0 International license.

To investigate the extent of codependency of the *dnaJ* and *dnaK* genes in Salmonella pathogenesis, we compared the competitive fitness of wild-type, Δ*dnaKJ*, and Δ*dnaKJ* Salmonella expressing the *dnaK* and/or *dnaJ* genes when inoculated as mixtures into the peritoneal cavity of C57BL/6 mice. About 100,000-fold fewer CFU of Δ*dnaKJ* Salmonella were recovered from livers and spleens of C57BL/6 mice than wild-type controls (Fig. 1B). Moreover, Δ*dnaKJ* Salmonella expressing *dnaK* or *dnaJ* alleles was nearly as attenuated as the parent Δ*dnaKJ* isogenic strain. In contrast, Δ*dnaKJ* Salmonella complemented with the *dnaKJ* operon became as virulent as wild-type controls (Fig. 1B and Fig. S1A). These investigations strongly suggest that DnaK and DnaJ mostly work together to promote virulence in this acute model of Salmonella infection.

### The DnaK-DnaJ chaperone system promotes antioxidant defense in Salmonella.

**FIG 2:**
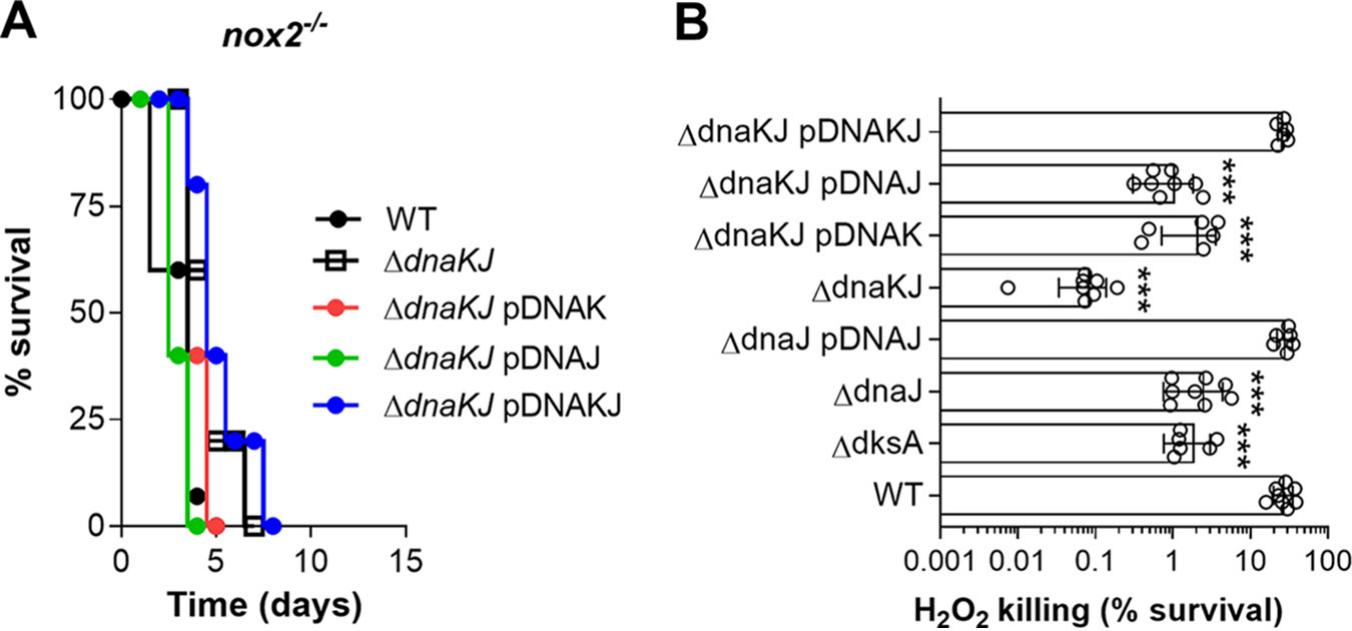
DnaK and DnaJ protect Salmonella from oxidative stress. (A) Survival of phagocyte NADPH oxidase-deficient *nox*2^−/−^ mice after i.p. challenge with ∼150 CFU of the indicated Salmonella strains. The data are from 8 to 10 mice in 2 independent experiments. (B) Survival of Salmonella grown overnight in LB broth after 2 h of treatment with 200 μM H_2_O_2_ in PBS. The data are the means ± standard deviations (SD) (*n* = 6) from 6 to 10 independent experiments. ***, *P < *0.001, as determined by one-way ANOVA compared to wild-type Salmonella.

Because DnaJ helps Salmonella resist the oxidative stress generated by the phagocyte NADPH oxidase in an acute model of salmonellosis (27), we studied whether DnaK participates in the antioxidant defense of this enteropathogen. We noted that Δ*dnaKJ* mutant Salmonella as well as Δ*dnaKJ* strains expressing the *dnaK* or *dnaJ* genes were as virulent as wild-type controls in *nox2*^−/−^ mice lacking the gp91*phox* subunit of the phagocyte NADPH oxidase (Fig. 2A). The attenuation of Δ*dnaKJ* Salmonella in NOX2-proficient mice is similar to that described for Δ*dksA* Salmonella (29). These findings strongly suggest that the DnaK/DnaJ chaperone system promotes resistance of Salmonella to oxidative stress engendered in the innate host response. We also noted that Δ*dnaKJ* Salmonella was extremely sensitive to H_2_O_2_ killing (Fig. 2B). Complementation of Δ*dnaKJ* Salmonella with the *dnaK* or *dnaJ* genes improved resistance to H_2_O_2_ to levels seen in Δ*dnaJ* or Δ*dksA* mutant Salmonella strains. Complementation of Δ*dnaKJ* Salmonella with a plasmid expressing both *dnaK* and *dnaJ* genes reestablished wild-type resistance to H_2_O_2_ killing (Fig. 2B). Together, these findings raise the possibility that DnaK and DnaJ genes may participate in the antioxidant defenses of Salmonella in DksA-dependent and -independent manners. Our investigations also suggest that DnaK and DnaJ promote resistance of Salmonella to oxidative stress in codependent and independent ways.

### The J domain of DnaJ promotes antioxidant defenses of Salmonella.

**FIG 3:**
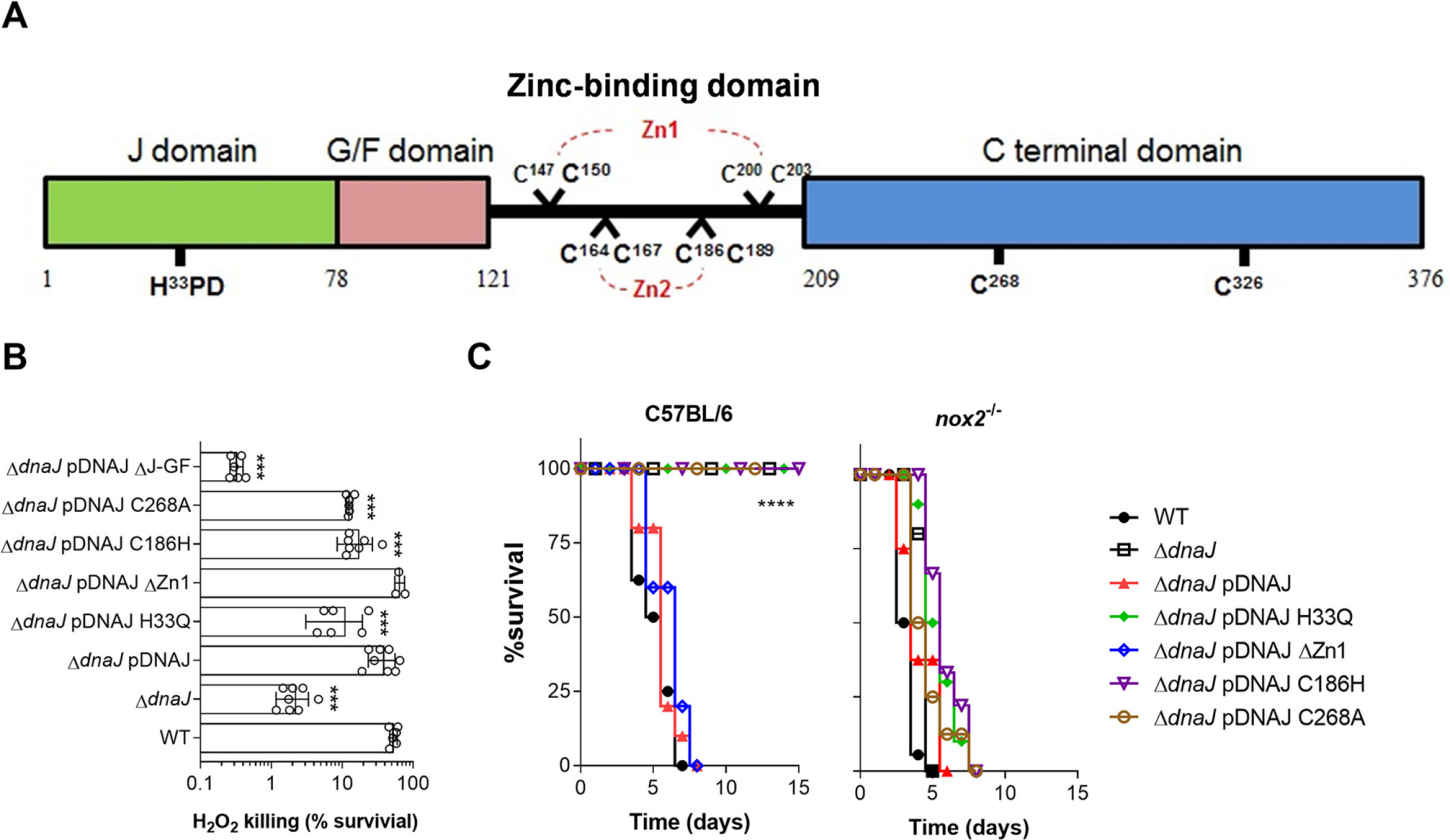
Impact of *dnaJ* variants in the antioxidant defenses of Salmonella. (A) Schematic representation of DnaJ regions consisting of the J, G/F (Gly/Phe-rich region), zinc-binding, and C-terminal domains. The J domain, via the evolutionarily conserved HPD motif, stimulates DnaK ATPase activity, and the G/F domain assists binding of DnaJ to DnaK. Four CXXCXGXG consensus sequences (where with C represents Cys, X is any other amino acid, and G is glycine) assemble into two zinc-binding domains, Zn1 and Zn2. The C-terminal domain contains 2 additional cysteine residues. (B) Susceptibility of wild-type and the indicated Salmonella mutants grown overnight in LB broth after 2 h of treatment with 200 μM H_2_O_2_ in PBS. The data are the means ± SD (*n* = 6 to 8) from 3 or 4 independent experiments. ***, *P < *0.001, as determined by one-way ANOVA compared to wild-type Salmonella. (C) C57BL/6 and *nox2^−^*^/−^ mice were i.p. inoculated with ∼150 CFU of the indicated Salmonella strains. Mouse survival was monitored over 2 weeks. The data are from 8 to 18 mice collected in 2 or 3 independent experiments. ****, *P < *0.0001, as determined by log-rank analysis compared to mice infected with wild-type Salmonella.

Our genetic analysis suggests that part of the role played by DnaJ in the antioxidant defenses of Salmonella relies on its cochaperone DnaK. The conserved HPD (His-Pro-Asp) motif in the J domain of DnaJ, in conjunction with the G/F domain (Fig. 3A), stimulates DnaK’s ATPase activity (1, 16). We examined whether DnaJ’s contribution to the antioxidant defenses of Salmonella is dependent on the J and G/F domains. Salmonella expressing a *dnaJ* allele lacking the J and G/F domains was as hypersusceptible to H_2_O_2_ as Δ*dnaJ* controls (*P = *0.9979 as assessed by one-way analysis of variance [ANOVA]) (Fig. 3B). Moreover, a strain expressing the *dnaJ* H33Q mutation in the J domain was hypersusceptible to H_2_O_2_ (Fig. 3B and Fig. S1B). Because the HPD motif in the J domain of DnaJ stimulates ATPase activity of DnaK (1, 5, 16), the susceptibility of Salmonella bearing the *dnaJ* H33Q allele suggests that at least part of the antioxidant defenses associated with DnaJ is dependent on DnaK. It should be noted that Δ*dnaJ* mutant Salmonella was as susceptible to H_2_O_2_ as an isogenic mutant expressing the *dnaJ* H33Q gene (*P = *0.2815) (Fig. 3B).

To identify domains of DnaJ that contribute to the resistance of Salmonella to oxidative stress, we engineered mutations in cysteine residues in the zinc-binding domain and C-terminal domain (CTD) of DnaJ. Salmonella strains harboring single (C147S or C203S), double (C147S C150S) or quadruple (C147S C150S C200S C203S, referred to as ΔZn1) substitutions in cysteine residues in the zinc 1-binding domain of DnaJ exhibited wild-type resistance to H_2_O_2_ killing (Fig. 3B and Fig. S1C). Consistent with published data (27), Salmonella strains expressing a C186H substitution at the zinc 2-binding domain or a C268A mutation at the CTD of DnaJ were more susceptible (*P < *0.001) to H_2_O_2_ than wild-type controls (Fig. 3B). These findings indicate that the J domain, zinc 2-binding domain, and CTD of DnaJ contribute to the antioxidant defenses of Salmonella. In addition, our findings suggest that the zinc 1-binding domain of DnaJ is dispensable for resistance of Salmonella to H_2_O_2_.

To investigate the contribution of DnaJ domains to resistance of Salmonella to oxidative stress generated by the phagocyte NADPH oxidase in the innate host response, C57BL/6 and *nox*2^−/−^ mice were inoculated with Salmonella strains expressing various *dnaJ* alleles. C57BL/6 mice survived infection with strains expressing Δ*dnaJ* or *dnaJ* H33Q, C186H, or C268A alleles. In contrast, the *dnaJ* ΔZn1 variant supported full Salmonella virulence (Fig. 3C). The Δ*dnaJ* mutant and Salmonella strains bearing *dnaJ* H33Q, C186H, or C268A alleles became as virulent as wild-type controls in immunodeficient *nox2*^−/−^ mice lacking the gp91*phox* subunit of the phagocyte NADPH oxidase (Fig. 3C). These findings suggest that the contribution of DnaJ to resistance to the phagocyte NADPH oxidase is mediated by the J-domain-dependent stimulation of DnaK’s ATPase activity as well as by oxidoreductase and holdase activities intrinsic to the zinc 2-binding domain and the CTD.

### DnaK and DnaJ minimize protein carbonylation in Salmonella experiencing oxidative stress.

**FIG 4:**
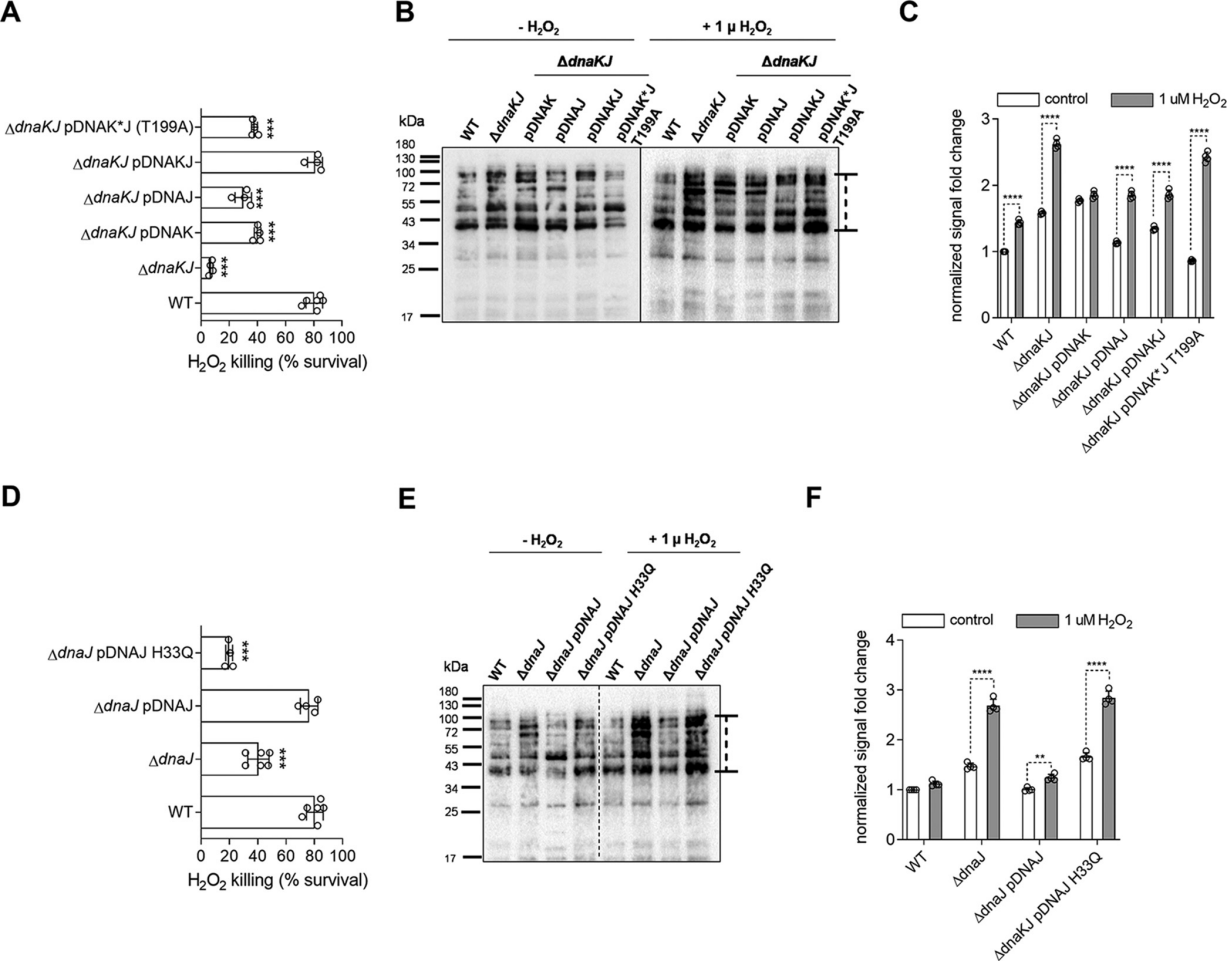
DnaK and DnaJ lessen protein carbonylation in anaerobic Salmonella exposed to H_2_O_2_. (A and D) Survival of anaerobic Salmonella exposed to 1 μM H_2_O_2_ for 2 h in an anaerobic chamber. ***, *P < *0.001, as determined by one-way ANOVA compared to wild-type Salmonella. The data are the means ± SD from 4 to 6 biological replicates collected in 2 or 3 days. (B and E) Protein carbonylation in anaerobic Salmonella treated with or without 1 μM H_2_O_2_ for 2 h was assessed by immunoblotting of DNP-derivatized proteins. The data are representative of 2 or 3 independent experiments. (C and F) Density of protein carbonylation was measured with ImageJ, and the density comparison region is indicated by a dotted line with a cap next to the blots. Fold change in density was calculated as density of mutant/density of wild-type controls. The data are the means ± SD (*n* = 4) from 4 independent measurements. ****, *P < *0.0001, as determined by one-way ANOVA.

We next examined possible mechanisms by which the DnaK/DnaJ chaperone system adds to the antioxidant defenses of Salmonella. Cells deficient in chaperones accumulate misfolded proteins, a situation that increases the risk of polypeptides becoming carbonylated (30, 31). Carbonylation of side chains of lysine, arginine, proline, and threonine residues can be detected by immunoblotting as 2,4-dinitrophenylhydraxine (DNPH)-derivatized products. To minimize endogenous oxidation of proteins by reactive oxygen species generated in aerobic metabolism, Salmonella strains were grown anaerobically for several days and then treated with or without 1 μM H_2_O_2_ for 2 h in an anaerobic chamber. The essential role played by the anaerobic master regulator FNR in resistance of Salmonella to the phagocyte NADPH oxidase suggests that, in some instances during infection, this intracellular pathogen must experience the antimicrobial activity of the respiratory burst under oxygen-limiting conditions (32). Similar to aerobic cultures (Fig. 2B), anaerobic Δ*dnaKJ*, Δ*dnaK*, and Δ*dnaJ* Salmonella strains showed hypersusceptibility when exposed to H_2_O_2_ in an anaerobic chamber (Fig. 4A). Proteins isolated from Δ*dnaKJ* Salmonella showed a 1.6-fold increase in protein carbonylation compared to wild-type controls (Fig. 4B and C). Proteins from single Δ*dnaK* or Δ*dnaJ* mutants exhibited less or similar carbonylation compared to control specimens isolated from Δ*dnaKJ* Salmonella (Fig. 4B and C). Exposure of Salmonella deficient in *dnaKJ*, *dnaK*, or *dnaJ* genes to H_2_O_2_ increased the level of carbonylated proteins compared to H_2_O_2_-treated wild-type controls (*P < *0.001). Moreover, treatment of a strain expressing the *dnaK* T199A allele with H_2_O_2_ also resulted in hypersusceptibility as well as high levels of carbonylated proteins (Fig. 4A to C and Fig. S1D). These results indicate that (i) DnaK and DnaJ prevent protein carbonylation in Salmonella exposed to H_2_O_2_ and (ii) DnaK’s ATPase is critical for this activity.

Increased susceptibility to H_2_O_2_ (Fig. 4D) and high levels of carbonylated proteins (Fig. 4E and F) were also detected in a Δ*dnaJ* Salmonella strain bearing a *dnaJ* H33Q allele. These findings suggest that the stimulation of DnaK’s ATPase activity by the J domain of DnaJ minimizes protein carbonylation in response to H_2_O_2_.

### DnaK and DnaJ activate the stringent response of H_2_O_2_-treated Salmonella.

**FIG 5:**
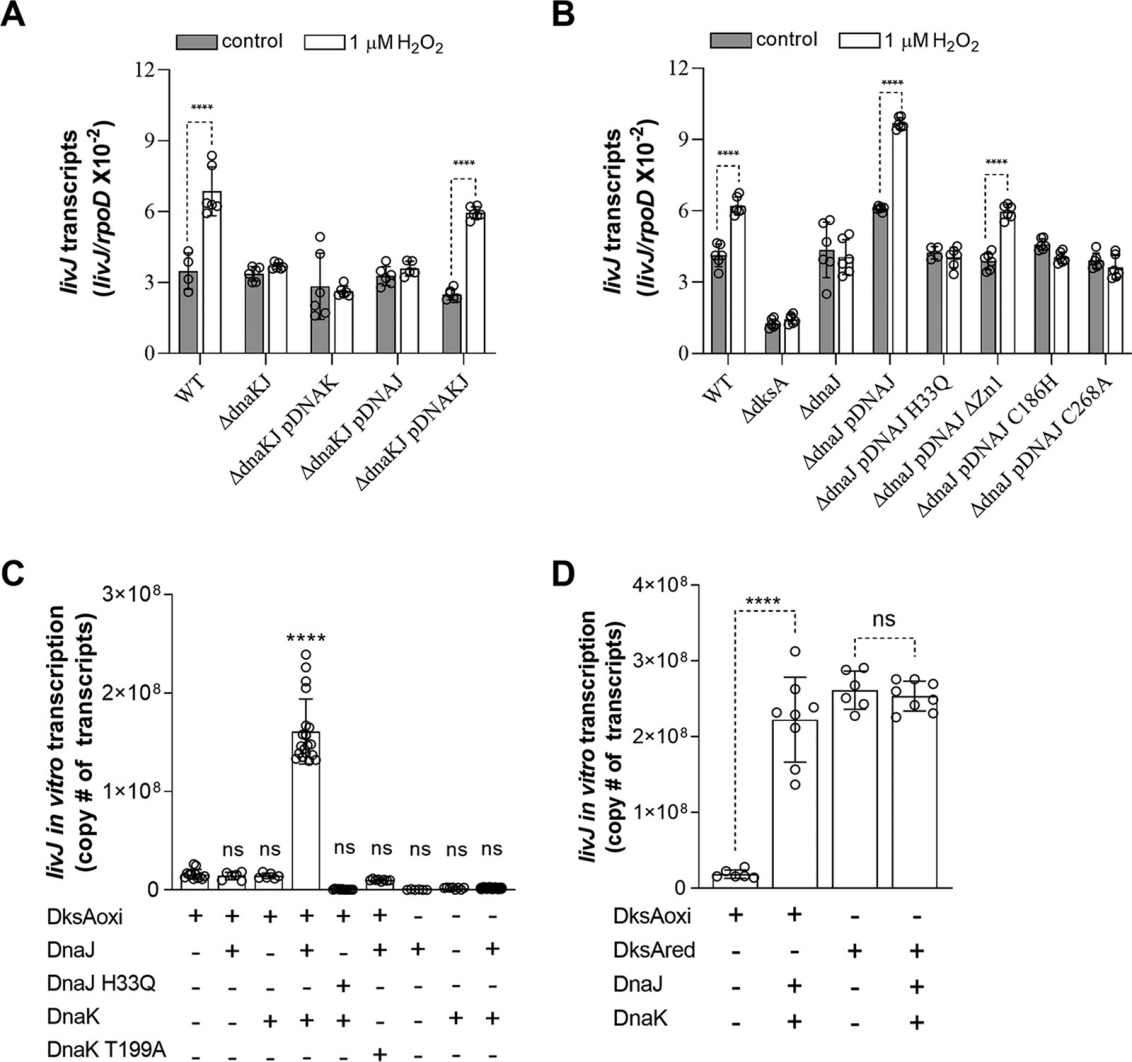
DnaK and DnaJ activate DksA-redox dependent gene expression. (A and B) The indicated Salmonella strains were grown in EGCA medium in an anaerobic chamber. Abundance of *livJ* transcripts in RNA isolated from anaerobic Salmonella treated with or without 1 μM H_2_O_2_ for 30 min was measured by qRT-PCR. The expression of the housekeeping gene *rpoD* was used as an internal control. The data are the means ± SD from 4 to 6 biological replicates collected in 2 or 3 independent days. ****, *P < *0.0001, as determined by one-way ANOVA. (C and D) Activation of *livJ* transcripts in *in vitro* transcription reaction mixtures containing 5 μM oxidized DksA, 50 nM DnaJ, and 500 nM DnaK proteins in the presence of RNA polymerase. Interactions of DnaJ and DnaK proteins with oxidized or reduced DksA are shown in panel D. Reduced DksA (5 μM) showed sufficiently activated *in vitro livJ* transcripts in the absence of DnaJ (50 nM) and DnaK (500 nM) proteins. The abundance of *livJ* transcripts was analyzed by qRT-PCR. The data are the means ± SD (*n* = 6 to 20) from at least 3 independent experiments. ****, *P < *0.0001, as determined by one-way ANOVA. ns, nonsignificant compared to reaction mixtures containing 5 μM oxidized DksA (C) or reduced DksA (D).

The regulator DksA coordinates transcriptional adaptations that increase fitness of Salmonella in an acute model of infection dominated by the antimicrobial activity of NOX2 (29, 33). Binding of oxidized DksA to RNA polymerase is facilitated by protein-protein redox interactions with DnaJ (27). Because DnaK and DnaJ work together to boost the antioxidant defenses of Salmonella (data herein), we investigated whether the activation of the stringent response in Salmonella undergoing oxidative stress reflects a cooperation between DnaK and DnaJ. We measured expression of *livJ* branched-chain amino acid transport and *hisG* histidine biosynthetic genes as proxies of the stringent response elicited upon contact of Salmonella with reactive oxygen species (27, 33). These investigations were performed under anaerobic conditions because a sizable fraction of DksA is oxidized in aerobically grown Salmonella (27). Salmonella grown anaerobically was treated with or without 1 μM H_2_O_2_. Δ*dnaKJ*, Δ*dnaK*, and Δ*dnaJ* Salmonella strains failed to activate *livJ* gene transcription in response to H_2_O_2_ (Fig. 5A and B). Complementation of Δ*dnaKJ* mutant Salmonella with the *dnaKJ* operon reestablished *livJ* transcription. As expected (27), the activation of *livJ* transcription elicited in response to H_2_O_2_ was blunted in Δ*dksA* Salmonella (Fig. 5B). In addition, Δ*dnaJ* Salmonella complemented with *dnaJ* H33Q, C186H, or C268A mutant alleles did not support *livJ* transcription in response to H_2_O_2_ (Fig. 5B). On the other hand, Salmonella bearing the *dnaJ* ΔZn1 allele induced as much *livJ* transcription in response to H_2_O_2_ as wild-type controls (Fig. 5B). Similar results were recorded when *hisG* transcription was analyzed (Fig. S2A and B). These findings indicate that DnaK and DnaJ cooperate in the stringent response of Salmonella undergoing oxidative stress. Our genetic inquiry also suggests that the allosteric activation of DnaK’s ATPase through interaction with the J domain of DnaJ positively regulates the stringent response of H_2_O_2_-treated Salmonella.

10.1128/mBio.03443-20.6 FIG S2 Transcription of *hisG* in Salmonella strains and titration of DnaK concentration of *in vitro* transcription. Download FIG S2, TIF file, 0.8 MB. Copyright © 2021 Kim et al. 2021 Kim et al. https://creativecommons.org/licenses/by/4.0/ This content is distributed under the terms of the Creative Commons Attribution 4.0 International license.

The cooperation of DnaK, DnaJ, and oxidized DksA seems to be at odds with the fact that DnaJ by itself can facilitate *livJ in vitro* transcription by oxidized DksA (27). Taking into account that the estimated concentration of DksA in E. coli is approximately 2 μM (22), we estimate that the intracellular concentration of DnaJ is about 50 nM (Fig. S2C). We report that 50 nM DnaJ fails to activate *livJ in vitro* transcription by oxidized DksA (Fig. 5C and D and Fig. S2D), which is in contrast to the activation seen in reaction mixtures containing 500 nM DnaJ (27). We then tested the effect that the addition of increasing concentrations of untagged DnaK has on *livJ in vitro* transcription by oxidized DksA, DnaJ, nucleoside triphosphates (NTPs), and RNA polymerase (Fig. S2D and E). A >10-fold induction of *livJ in vitro* transcription was noted upon the addition of 500 nM DnaK to reaction mixtures containing oxidized DksA, DnaJ, and RNA polymerase (Fig. 5C and D). The recombinant DnaK T199A variant, which lacks a critical threonine in the ATPase catalytic site and shows normal interaction with DnaJ in biochemical *in vitro* pulldown assays (Fig. S2F and G), and the DnaJ H33Q recombinant variant, which does not support DnaK’s ATPase activity (34), failed to induce *livJ in vitro* transcription (Fig. 5C). DnaK or DnaJ proteins by themselves and in combination failed to activate *livJ in vitro* transcription (Fig. 5C). The DnaK/DnaJ couple also failed to enhance the transcription of *livJ* supported by reduced DksA (Fig. 5D). These investigations demonstrate that at biologically relevant concentrations, the loading of oxidized DksA onto RNA polymerase by DnaJ is facilitated by its DnaK chaperone partner.

### DnaJ, not DnaK, directly binds to oxidized DksA.

**FIG 6:**
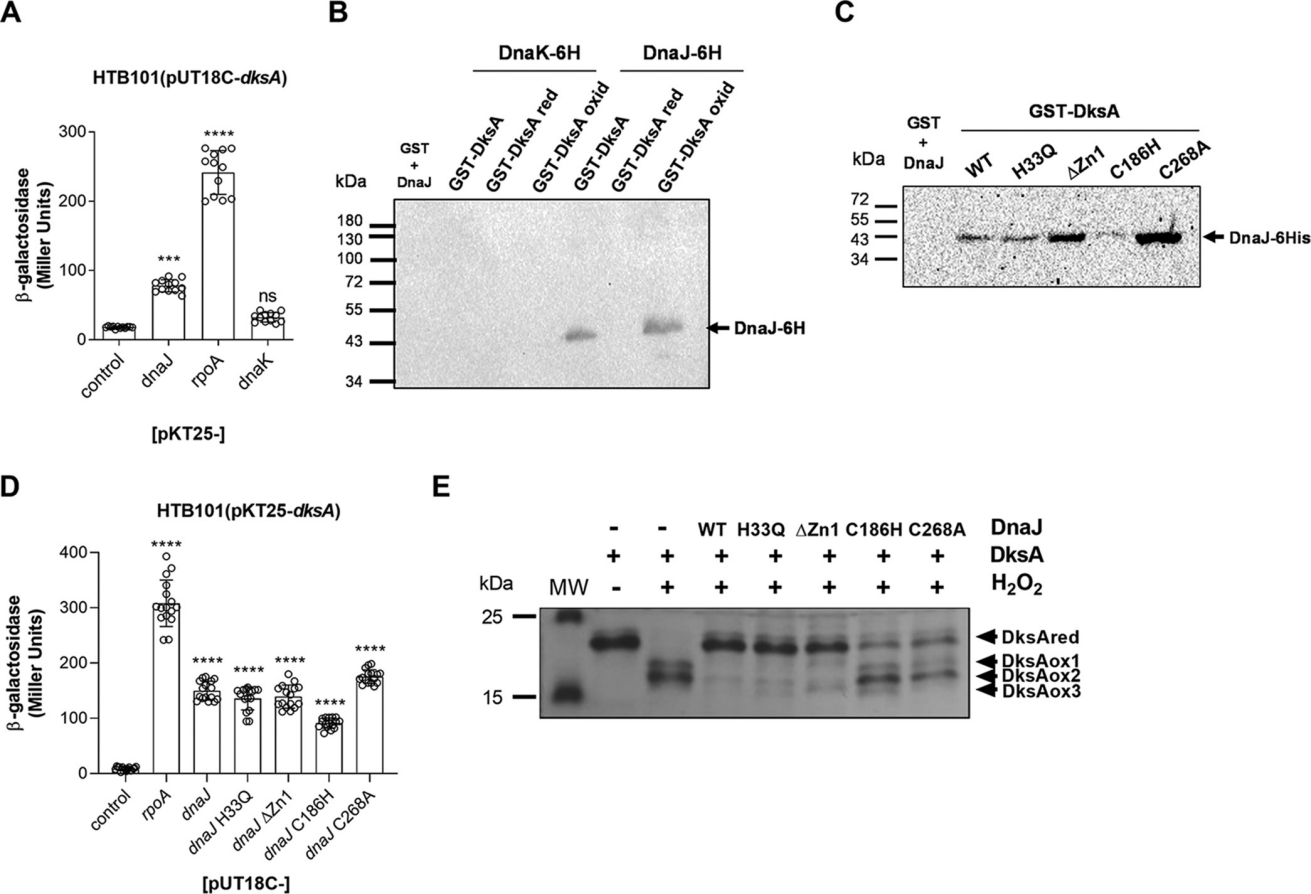
Interactions of DnaK and DnaJ proteins with DksA. Interactions of DnaK and DnaJ proteins with DksA were evaluated by a bacterial two-hybrid system (A and D) and biochemical pulldown assays (B and C). The data in panel A are the means ± SD from 12 to 16 biological replicates from 3 or 4 independent experiments ***, *P < *0.001, and ****, *P < *0.0001, as determined by one-way ANOVA. ns, nonsignificant compared to the negative vector control. Blots in panels B and C are representative of 3 or 4 independent experiments. (E) Reduced DksA (5 μM) residues were alkylated with AMS, and the proteins were visualized on nonreducing SDS-PAGE gels stained with Coomassie brilliant blue. Samples were treated with or without 500 μM H_2_O_2_ at 37°C for 1 h in the presence and absence of 5 μM DnaJ variants. The data are from 2 or 3 independent experiments.

To further examine the critical role DnaK plays in the activation of the stringent response, we used a bacterial two-hybrid system that reconstitutes interactions of the T18 and T25 subunits of adenylate cyclase. A T18-DksA fusion showed significant binding to the T25-DnaJ construct (Fig. 6A). In sharp contrast, the T18-DksA and T25-DnaK pair exhibited as poor binding as the combination of the T18-DksA and the T25 negative vector control (Fig. 6A). The pair T18-DksA and T25-RpoA, a subunit of RNA polymerase, served as a positive control (Fig. 6A). Because the T25 N-terminal fusion could interfere with DnaK’s binding performance, we conducted biochemical pulldown assays to independently test binding of C-terminal DnaK- and DnaJ-His-tagged proteins to DksA. These investigations showed that oxidized DksA, not the reduced protein, directly interacts with DnaJ (Fig. 6B and Fig. S3A). In sharp contrast, oxidized DksA did not interact with DnaK (Fig. 6B). The DnaK protein also failed to bind to DksA in buffer containing K^+^, Mg^2+^, and ATP (Fig. S3B). These findings suggest that DnaK does not bind directly to DksA irrespectively of the redox state of the latter and provide a reasonable explanation for the inability of DnaK alone to support the DksA-dependent activation of *livJ* transcription (Fig. 5A and C). We also observed that a DnaJ variant bearing the H33Q mutation in the J domain still binds to DnaK using a bacterial two-hybrid system (Fig. S3C) and a biochemical pulldown assay (Fig. S3D and E), indicating that the failure of the DnaJ H33Q variant to stimulate the DnaK- and DksA-dependent stringent response in H_2_O_2_-treated Salmonella cannot be explained by its inability to bind to DnaK. These investigations also suggest that the activation of DnaK’s ATPase activity through interactions with the HPD motif of DnaJ is essential for the stimulation of the stringent response in H_2_O_2_-treated Salmonella.

10.1128/mBio.03443-20.7 FIG S3 Characterization of DnaK binding to DnaJ H33Q and determination of zinc content of recombinant DnaJ variants. Download FIG S3, TIF file, 1.4 MB. Copyright © 2021 Kim et al. 2021 Kim et al. https://creativecommons.org/licenses/by/4.0/ This content is distributed under the terms of the Creative Commons Attribution 4.0 International license.

Having established that oxidized DksA binds to DnaJ but not DnaK (Fig. 6B), we examined the domains of DnaJ responsible for binding to DksA. DnaJ variants bearing H33Q, ΔZn1, or C268A substitutions interacted with DksA with an affinity apparently similar to or greater than that of the wild-type DnaJ protein control (Fig. 6C; Fig. S3F shows input proteins for the pulldown in Fig. 6C). The wild type and DnaJ H33Q, C186H, and C268A variants harbored about 2 molar equivalents of zinc, which contrasts with the equimolar concentrations of zinc bound to DnaJ ΔZn1 (Fig. S3G). A DnaJ C186H protein variant showed slightly reduced binding to DksA compared to wild-type controls (Fig. 6C), a finding that is consistent with our previously published work (27). The binding of DnaJ variants bearing H33Q, ΔZn1, C186H, or C268A substitutions to DksA was independently evaluated using a bacterial two-hybrid system. The T18-DnaJ H33Q construct supported excellent reconstitution of β-galactosidase activity when combined with the T25-DksA plasmid (Fig. 6D). The T25-DksA and T18C-DnaJ variants bearing ΔZn1 or C268A substitution pairs exhibited high levels of binding, whereas the combination of T25-DksA and T18C-DnaJ C186H showed poor interaction compared to wild-type controls (*P < *0.0001) (Fig. 6D). Collectively, these investigations suggest that the inability of the *dnaJ* H33Q variant to activate an H_2_O_2_-dependent stringent response cannot be explained by an inability to bind to DksA.

In addition to exhibiting holdase activity, DnaJ reduces two disulfide bonds in oxidized DksA (27). We tested the oxidoreductase activity of the DnaJ H33Q variant (Fig. 6E). These experiments showed that the DnaJ H33Q protein retains excellent oxidoreductase activity for oxidized DksA. Similar results were seen when the DnaJ ΔZn1 variant was tested. As reported previously (27), DnaJ C186H and DnaJ C268A protein variants were unable to reduce oxidized DksA, supporting the idea that cysteine residues in the zinc 2-binding domain and CTD participate in the oxidoreductase activity of DnaJ. Together, these investigations suggest that the inability of the DnaJ H33Q protein to promote Salmonella virulence, antioxidant defense, and DksA-mediated transcription occurs despite its excellent oxidoreductase activity.

### DksA cooperates with DnaK and DnaJ in Salmonella pathogenesis but not thermotolerance or motility.

**FIG 7:**
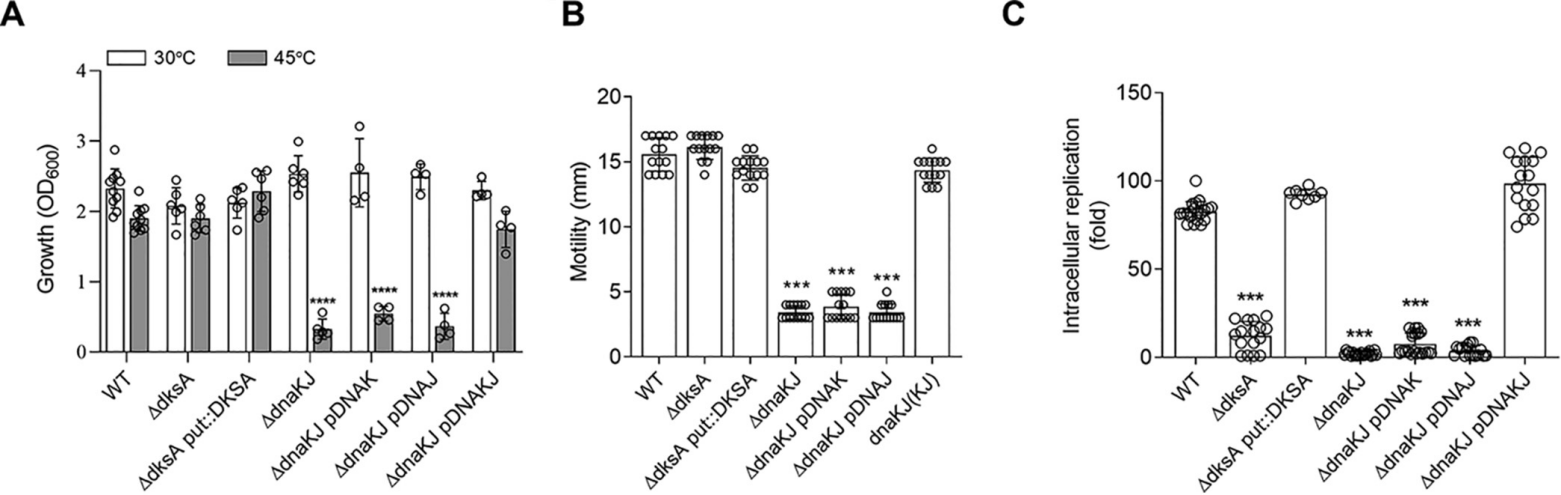
Contribution of *dnaK*, *dnaJ*, and *dksA* to thermotolerance, motility, and pathogenesis of Salmonella. (A) Growth of the indicated Salmonella strains in LB broth for 6 h at 30°C or 45°C in a shaker incubator. The data are the means ± SD (*n* = 4 to 10) from 4 to 8 independent experiments. (B) Motility was assessed by spotting 10^7^ CFU of the indicated Salmonella strains in 0.3% LB agar plates for 3 h at 37°C. The swimming zone was measured in millimeters. The data are the means ± SD (*n* =14) from at least 4 independent experiments. (C) Intracellular replication of Salmonella in J774 cells 18 h postinfection was determined by CFU measurement. The data are the means ± SD (*n* = 8 to 20) from at least 3 independent experiments. ***, *P < *0.001, and ****, *P < *0.0001, as determined by one-way ANOVA.

The investigations described above indicate that by interacting with oxidized DksA, the DnaK/DnaJ chaperone system regulates transcriptional signatures characteristic of the stringent response in H_2_O_2_-treated Salmonella. It is therefore possible that some of the functions ascribed to the DnaK/DnaJ chaperone couple could be mediated through DksA-dependent transcriptional programs. Thus, we studied the thermotolerance, motility, and intracellular growth of Salmonella strains deficient in *dnaK*, *dnaJ*, or *dksA* genes. A Δ*dksA* Salmonella strain grew at high temperatures as well as wild-type controls (Fig. 7A) and sustained excellent swimming activity as well (Fig. 7B). In contrast, Δ*dnaK* and Δ*dnaJ* mutant Salmonella strains exhibited temperature sensitivity and lost swimming ability (Fig. 7A and B). On the other hand, lack of either *dksA*, *dnaK*, or *dnaJ* genes resulted in similar intracellular growth defects of Salmonella in J774 macrophage-like cells (Fig. 7C). Deficiency of intracellular growth of Salmonella caused by deficient *dnaK*, *dnaJ*, or *dksA* genes was reversed by complementation with the *dnaKJ* operon or the *dksA* gene, respectively (Fig. 7C). Salmonella strains which blocked stimulation of DnaK’s ATPase activity by the *dnaJ* H33Q or the *dnaK* T199A alleles were hypersensitive to heat, sustained lower swimming ability and showed growth defects in J774 macrophage (Fig. S4A to C). These investigations suggest that DksA, DnaK, and DnaJ work together in Salmonella pathogenesis but not in thermotolerance and motility. This research also indicates that DnaK’s ATPase activity through the HPD domain of DnaJ is essential for thermotolerance, motility, and Salmonella pathogenesis.

10.1128/mBio.03443-20.8 FIG S4 Effect of DnaJ H33Q and DnaK T199A mutations on thermotolerance, motility, and pathogenesis of Salmonella. Download FIG S4, TIF file, 0.5 MB. Copyright © 2021 Kim et al. 2021 Kim et al. https://creativecommons.org/licenses/by/4.0/ This content is distributed under the terms of the Creative Commons Attribution 4.0 International license.

## DISCUSSION

**FIG 8:**
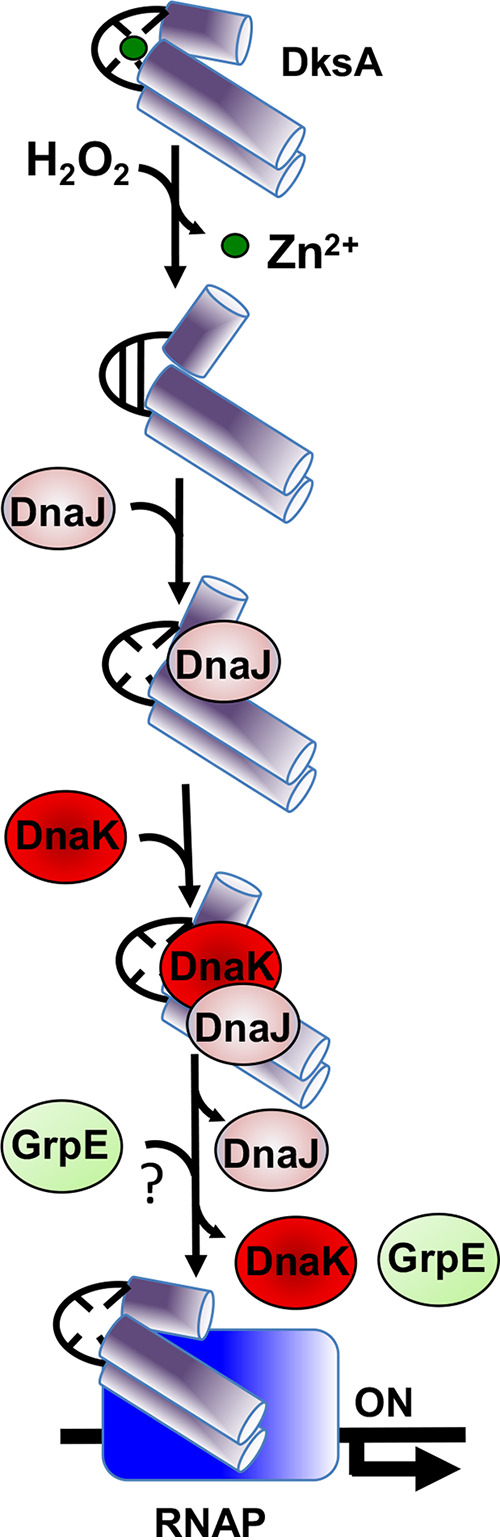
Model for the DnaK/DnaJ-mediated assembly of a DksA-RNA polymerase (RNAP) complex in response to reactive oxygen species. Hydrogen peroxide (H_2_O_2_) induces disulfide formation and zinc release in the transcriptional regulator DksA. DnaJ recognizes misfolded, disulfide-bonded DksA as a client protein. The oxidoreductase activity of DnaJ reduces the disulfide bonds in DksA, transferring the reduced, unfolded DksA protein to ATP-bound DnaK. The J domain of DnaJ stimulates ATPase activity of DnaK, which firmly binds to unfolded DksA. Although not proven in our investigations, it is possible that the nucleotide exchange factor GrpE facilitates refolding of DksA by DnaK. Together, the DnaK/DnaJ/GrpE chaperone system enables the formation of RNA polymerase-DksA complexes in response to oxidative stress.

The stringent response aids the adaptation of bacteria to stressful environmental conditions. In Gram-negative bacteria, this genetic program is regulated by the coordinated actions that the DksA protein and the nucleotide alarmone (p)ppGpp exert on the kinetics of the transcription open complex (35). Given that its concentrations are remarkably constant in the cell under various growth conditions (36, 37), how the DksA protein is selectively loaded into the secondary channel of RNA polymerase under starvation and stress conditions has been puzzling. Our present and past investigations indicate that loading of oxidized DksA to RNA polymerase is aided by DnaJ (27). Cysteine residues in the globular domain of DksA are oxidized by reactive oxygen and nitrogen species (27, 33). Disulfide-bonded DksA molecules are recognized by the DnaJ cochaperone, whose oxidoreductase and holdase activities facilitate loading of DksA into RNA polymerase, activating amino acid biosynthesis gene expression in Salmonella undergoing oxidative stress (27). Investigations shown herein demonstrate that DnaJ’s oxidoreductase and foldase activities are not sufficient to assemble RNA polymerase-DksA complexes *in vitro* or *in vivo* in the absence of DnaK. Accordingly, in the absence of either DnaK or DnaJ, H_2_O_2_-treated Salmonella fails to activate *hisG* or *livJ* gene transcription. This conclusion is further substantiated by the observation that together DnaK and DnaJ regulate the transcriptional activity of oxidized DksA in an *in vitro* transcription system. Our investigations are consistent with the idea that DnaJ/DnaK refold oxidized DksA, thus facilitating the interaction of this transcription factor with RNA polymerase (Fig. 8). Although not tested experimentally, it is likely that the nucleotide exchange factor GrpE enables the release and folding of DksA from DnaK, as recently described for the multidomain protein firefly luciferase *in vitro* (38).

The DksA regulator was identified in a genetic screen for loci that complemented heat shock defects of a *dnaKJ* mutant E. coli strain (13). The authors speculated that “some of the *dnaK* mutant phenotypes are due to lower expression of *dksA*.” According to this idea, *dksA* mRNA levels were increased upon the overexpression of *dksA* in Δ*dnaK* E. coli (13). The overexpression of *dksA* in the suppressor mutant appears to be driven by a transcript originating upstream of the major *dksA* promoter (39). The overexpression of DksA has been hypothesized to activate the transcription of an unidentified product whose promoter is directly or indirectly regulated by artificially elevated DksA concentrations (13, 39). The differential thermosensitivity of Δ*dnaK*, Δ*dnaJ*, and Δ*dksA* Salmonella strains is in line with the latter model, and suggest that elevated DksA levels reverse some of the phenotypes associated with *dnaKJ* mutations indirectly. In E. coli, rescuing of the thermosensitivity associated with the Δ*dnaKJ* mutation by the overexpression of DksA has recently been shown to be dependent on polyphosphate synthesis (40). Kang and Craig entertained the possibility that under some conditions DnaK and DksA may work together (13). Our investigations demonstrate that DnaK, DnaJ, and DksA form a functional complex that activates the transcriptional program known as the stringent response in Salmonella experiencing oxidative stress (Fig. 8). In comparison with its interactions with DnaJ, DksA appears to interact weakly or transiently with DnaK as suggested by the lack of reconstitution of a bacterial two-hybrid system between pT18-DksA and pT25-DnaK constructs. These observations are consistent with our previously published tandem-affinity purification (TAP) approaches, which identified interactions of DksA with DnaJ but not with DnaK (27). Nonetheless, the transient interactions of DnaK with DnaJ/DksA complexes allow the formation of transcriptionally active RNA polymerase in response to oxidative stress.

Zinc-starving Pseudomonas replaces the expression of a zinc-replete C4 DksA protein with a zinc-lacking C2 DksA paralogue (41, 42), suggesting that the metalation of DksA is susceptible to the levels of zinc in the environment. Because the zinc cation plays a structural role in C4 DksA homologues (43), a fraction of DksA could be present as an apoprotein in bacterial cells starved for zinc. Partially unfolded, demetalated DksA apoproteins in zinc-starved bacteria may become clients of the DnaK/DnaJ chaperone system. Future experiments will be needed to determine if the DnaK/DnaJ chaperone system regulates loading of DksA into RNA polymerase in response to stimuli such as zinc starvation.

Our investigations are consistent with a model in which oxidation of thiol groups in the globular domain of DksA triggers partial unfolding of this transcriptional factor (27). Thiol oxidation of DksA cysteines rather than carbonylation of amino acid residues must signal the interaction of oxidized DksA with DnaJ, as suggested by the fact that the addition of the thiol-reducing agent dithiothreitol (DTT) reversed the binding of oxidized DksA to DnaJ (27). The partial unfolding of DksA elicited upon exposure to reactive oxygen species signals binding to DnaJ. Cysteine residues in the Zn2-binding domain and CTD of DnaJ exhibit holdase and oxidoreductase activities against oxidized DksA. Our investigations herein indicate that DnaJ presents the reduced DksA protein to DnaK. This interaction involves the G/F and zinc-binding domains of DnaJ, as shown previously for other clients (9, 44). In the absence of DnaJ, DnaK is unable to bind to oxidized DksA. Independently of delivering DksA to DnaK, DnaJ’s HPD motif must trigger ATPase activity of its cochaperone, as suggested by the fact that a DnaJ H33Q mutant lacking a key residue in the J domain and a DnaK variant bearing the T199A mutation at the ATPase catalytic site are equally unable to stimulate DksA-RNA polymerase complex formation in response to H_2_O_2_.

Our investigations demonstrate that the DnaK/DnaJ chaperone system is critically important for the antioxidant defenses of Salmonella. Aerobic and anaerobic Salmonella strains bearing deletions in *dnaK* or *dnaJ* are hypersusceptible to the antimicrobial activity of H_2_O_2_. This observation is consistent with published data that showed that DnaK and DnaJ protect E. coli and Salmonella, respectively, against H_2_O_2_ killing (27, 45). Most of the antioxidant defenses associated with this chaperone system are codependent on DnaK and DnaJ. However, these heat shock proteins can also independently antagonize oxidative stress. For example, DnaJ’s oxidoreductase, holdase, and foldase activities by themselves may contribute to the antioxidant defenses of Salmonella (27). On the other hand, DnaK can form a functional chaperone system with the DnaJ-like proteins CbpA and DjlA (46–48). The nucleotide exchange factor GrpE facilitates recycling of DnaK-ADP to DnaK-ATP, thus aiding the folding of client proteins (38). It is quite possible that the DnaK/DnaJ-dependent antioxidant defenses described herein involve GrpE. Unfortunately, we were not able to test this hypothesis, because despite multiple attempts, we were unable to generate a Δ*grpE* Salmonella strain. This suggests that *grpE* is essential in *dnaK*^+^ *dnaJ*^+^ Salmonella, as described for E. coli (49).

DnaK has previously been shown to mediate antioxidant defense in E. coli. We now demonstrate that the antioxidant defenses associated with DnaK mediate resistance of Salmonella against the oxidative burst of the phagocyte NADPH oxidase. The critical role DnaK plays in resistance to NOX2-dependent antimicrobial activity likely stems from the pleotropic functions of this chaperone. By shortening the dwelling time proteins spend in the unfolded state, the DnaK/DnaJ chaperone system minimizes the risk of metal-catalyzed carbonylation of the side chains of proline, lysine, arginine, and threonine residues (31, 50–52). DnaK may add to the antioxidant defenses and Salmonella pathogenesis by other means as well. For example, the DnaK/DnaJ-dependent regulation of branched-chain amino acid biosynthesis could play important roles in Salmonella pathogenesis (19). Moreover, E. coli cells bearing a mutation in *dnaK* favor the Entner-Doudoroff pathway (53). Oxidative phosphorylation satisfies most energetic needs of cells using the Entner-Doudoroff pathway (52). Because overutilization of the respiratory chain over glycolysis predisposes to oxidative stress (54), a preference for respiration that follows the utilization of the Entner-Doudoroff pathway may contribute to the sensitivity of Δ*dnaKJ* mutants to oxidative stress. In addition, our investigations demonstrate that DnaK is critical for the H_2_O_2_-dependent activation of the stringent response, a genetic program previously associated with antioxidant defenses (27, 29).

The antioxidant defenses associated with the DnaK/DnaJ cochaperone system protect Salmonella against the respiratory burst sustained by the phagocyte NADPH oxidase. It will be interesting to evaluate the extent to which DnaK-regulated resistance to oxidative stress contributes to the virulence ascribed to this chaperone in diverse bacterial pathogens such as Brucella suis, Mycobacterium tuberculosis, Staphylococcus aureus, Streptococcus mutans, Campylobacter, and *Helicobacter* (55). However, the contribution of DnaK to bacterial pathogenesis is unlikely to be limited to resistance to oxidative stress. For example, DnaK optimizes expression of invasion programs in Salmonella (56).

## MATERIALS AND METHODS

### Bacterial strains, plasmids, and growth conditions.

The derivatives of Salmonella enterica serovar Typhimurium strain 14032s and Escherichia coli, as well as the plasmids used in this study, are listed in Tables S1 and S2. Deletion mutants were constructed using the λ-Red homologous recombination system (57). *Pfu* ultra-high-fidelity DNA polymerase (Agilent, Santa Clara, CA) was used to perform genetic mutagenesis. Bacteria were grown in LB broth and E salts medium [0.2% MgSO_4_, 2% C_6_H_8_O_7_·H_2_O, 10% K_2_HPO_4_, 3.5% Na(NH_4_)HPO_4_·4H_2_O] supplemented with 0.1% Casamino Acids and 4% d-glucose (pH 7.0) (EGCA) (41) at the permissible temperatures 30°C or 37°C. Penicillin, chloramphenicol, and kanamycin were added at the final concentrations of 250, 40, and 50 μg/ml, respectively.

10.1128/mBio.03443-20.2 TABLE S1 Bacterial strains used in this study. Download Table S1, DOC file, 0.05 MB. Copyright © 2021 Kim et al. 2021 Kim et al. https://creativecommons.org/licenses/by/4.0/ This content is distributed under the terms of the Creative Commons Attribution 4.0 International license.

10.1128/mBio.03443-20.3 TABLE S2 Plasmids used in this study. Download Table S2, DOC file, 0.06 MB. Copyright © 2021 Kim et al. 2021 Kim et al. https://creativecommons.org/licenses/by/4.0/ This content is distributed under the terms of the Creative Commons Attribution 4.0 International license.

### Animal studies.

For survival assays, 6- to 8-week-old C57BL/6 mice and congenic *nox*2*^−/−^* mice deficient in the gp91*phox* membrane subunit of the phagocyte NADPH oxidase (58) were inoculated intraperitoneally (i.p.) with ∼150 CFU of Salmonella grown overnight in LB broth. Mouse survival was monitored on a daily basis over 2 weeks. Salmonella-infected mice showing signs of disease were humanely euthanized via CO_2_ inhalation and cervical dislocation. Percent survival was calculated as follows: [(number alive at the end of that day)/(number alive at the beginning of that day)] × 100. For competitive assays, 6- to 8-week-old C57BL/6 mice were inoculated i.p. with ∼1,000 CFU of a mixture containing equal numbers of two strains of Salmonella. The bacterial burden was quantified in livers and spleens 4 days postinfection by plating on LB agar containing the appropriate antibiotics. Competitive index was calculated as follows: (strain 1/strain 2)_output_/(strain 1/strain 2)_input_. All mice were used according to protocols approved by the Institutional Animal Care and Use Committee (IACUC) at the University of Colorado School of Medicine.

### Susceptibility to H_2_O_2_.

Stationary-phase Salmonella organisms grown in LB broth at 30°C were diluted in phosphate-buffered saline (PBS) to a final concentration of 10^6^ CFU/ml. The samples were treated with or without 200 μM H_2_O_2_ at 37°C for 2 h. Where indicated, anaerobic cultures were challenged with 1 μM H_2_O_2_ for 2 h. The percent surviving bacteria was calculated as follows: [(CFU from H_2_O_2_-treated sample/CFU from untreated sample)] × 100.

### Protein carbonylation.

To minimize endogenous oxidation of proteins generated under aerobic conditions, Salmonella cultures were passaged for 10 days at 30°C in LB broth in a Bactron anaerobic chamber (Shel Lab, Cornelius, OR) containing 5% hydrogen, 5% carbon dioxide, and 90% nitrogen. The bacterial cultures were diluted in PBS to an optical density at 600 nm (OD_600_) of 0.3 in an anaerobic chamber, and the specimens were treated with or without 1 μM H_2_O_2_ at 30°C for 2 h. The cultures were harvested by centrifugation under anaerobic conditions and resuspended in PBS containing 1 mM DTT. Samples were disrupted by sonication with 40% amplitude for 4 s under aerobic conditions, and protein concentration was determined with the bicinchoninic acid (BCA) protein assay kit (Pierce, Rockford, IL). The carbonyl groups in amino acid side chains were derivatized to 2,4-dinitrophenylhydrazone (DNP-hydrazone) by reaction with 2,4-dinitrophenylhydrazine (DNPH) supplied by the Oxyblot protein oxidation detection kit (Millipore Sigma, St. Louis, MO). Two micrograms of DNP-derivatized protein samples was subjected to 12% SDS-PAGE followed by immunoblotting using a 1:2,000 dilution of anti-DNP antibody (Millipore Sigma, St. Louis, MO), followed by a 1:10,000 dilution of goat anti-mouse IgG conjugated with horseradish peroxidase (Pierce). Blots were visualized using the ECL Prime Western blotting detection system supplied by GE (GE Healthcare Life Sciences, Marlborough, MA). Density of protein carbonylation was measured by the ImageJ program provided by NIH. After challenge with 1 μM H_2_O_2_ for 2 h under anaerobic conditions, the percent surviving bacteria was enumerated by plating on LB agar and incubated overnight in an anaerobic chamber. Percent survival was calculated as follows: [(CFU from H_2_O_2_-treated sample/CFU from untreated sample)] × 100.

### RNA isolation and quantitative RT-PCR.

Salmonella grown overnight in LB broth at 30°C in a BACTRON anaerobic chamber were subcultured 1:100 in EGCA medium. Cells grown to an OD_600_ of 0.3 were treated with or without 1 μM H_2_O_2_ at 30°C for 30 min. Total RNA was isolated using the High Pure RNA isolation kit (Roche, Indianapolis, IN). The synthesis of cDNA was achieved using 1 μg of purified RNA, TaqMan gene expression master mix (Thermo Fisher Scientific, Grand Island, NY), and N6 random primers (Thermo Fisher Scientific). Quantitative RT-PCR was performed using specific primers and probes (Table S3) containing 5′,6-carboxyfluorescein and 3′ black hole quencher 1 modification in a CFX Connect real-time system (Bio-Rad, Hercules, CA). PCR-amplified DNA fragments containing the gene of interest were used to generate standard curves. The abundance of transcripts within each sample was normalized to internal transcripts of the housekeeping gene *rpoD*.

10.1128/mBio.03443-20.4 TABLE S3 Oligonucleotides used in this study. Download Table S3, DOC file, 0.08 MB. Copyright © 2021 Kim et al. 2021 Kim et al. https://creativecommons.org/licenses/by/4.0/ This content is distributed under the terms of the Creative Commons Attribution 4.0 International license.

### Overexpression and purification of proteins.

*dksA*, *dnaK*, and its point mutant were cloned into BamHI and XhoI sites of the glutathione *S*-transferase (GST) fusion plasmid pGEX-6P-1 (GE Healthcare Life Sciences). Full-length *dnaK*, *dnaJ*, and its point mutants were cloned as C-terminal 6×His fusions into NdeI and XhoI sites of the pET-22b(+) plasmid (Novagen, Madison, WI). All constructs and mutations were confirmed by sequence analysis. Plasmids were expressed in E. coli BL21(DE3) (Thermo Fisher Scientific) (Table S1). Briefly, cells grown in LB broth at 37°C to an OD_600_ of 0.5 to 0.8 were treated with 0.1 mM isopropyl-β-d-thiogalactopyranoside. After 3 h, the cells were harvested, disrupted by sonication, and centrifuged to obtain cell extracts. GST and 6×His-tagged fusion proteins were purified using glutathione-Sepharose 4B (bioWORLD, Dublin, OH) and TALON metal-affinity chromatography (Clontech, Mountain View, CA), respectively, according to manufacturer’s protocols.

To perform *in vitro* transcriptional assays, the GST tags were removed from GST-DksA and GST-DnaK variant proteins bound to a glutathione-Sepharose 4B resin. PreScission protease was added to recombinant GST-DksA, GST-DnaK, or GST-DnaK T199A proteins in PBS containing 10 mM DTT. After overnight incubation at 4°C, untagged proteins were eluted with PBS. For further purification of DksA protein, size exclusion chromatography on Superdex 75 (GE Healthcare Life Sciences) was used. Purified DksA proteins were aliquoted inside a BACTRON anaerobic chamber (Shel Lab). The purity and mass of the recombinant proteins were assessed by SDS-PAGE.

### *In vitro* transcription.

*In vitro* transcription reactions were quantified by quantitative reverse transcription-PCR (qRT-PCR) as previously described (27, 59). Briefly, oxidized or reduced DksA proteins were prepared by treatment with 1 mM H_2_O_2_ or 1 mM DTT in 40 mM HEPES, pH 7.4, at 37°C for 1 h. Excess H_2_O_2_ or DTT was removed using an Amicon Ultra-10 centrifugal filter (Millipore Sigma, St. Louis, MO). Transcription reactions were performed in 40 mM HEPES (pH 7.4), 2 mM MgCl_2_, 60 mM potassium glutamate, 0.1% Nonidet P-40, a 200 μM concentration each of ATP, GTP, CTP, and UTP (Thermo Fisher Scientific), 8 U RiboLock RNase inhibitor (Thermo Fisher Scientific), 1 nM *livJ* DNA templates (Table S2), 5 nM E. coli holoenzyme RNA polymerase (New England Biolabs, Ipswich, MA), and 5 μM oxidized or reduced DksA proteins. Where indicated, the reaction mixtures contained 50 nM DnaJ variants in the presence and absence of 500 nM tag-free DnaK variant proteins. Reaction mixtures were incubated at 37°C for 10 min and then heat inactivated at 70°C for 10 min. After DNase I treatment, template DNA was removed from the reactions with a DNA-free DNA removal kit (Thermo Fisher Scientific, Grand Island, NY), and the resulting RNA was used as the template to generate cDNA using 100 U Moloney murine leukemia virus (MMLV) reverse transcriptase (Promega, Madison, WI), 0.45 μM N6 random hexamer primers (Thermo Fisher Scientific) and 20 U RNase inhibitor (Promega). The amount of cDNA synthesized following 1 h of incubation at 42°C was quantified by qRT-PCR using gene-specific primers and probes (Table S3). Specific transcripts were normalized to standard curves using known transcript concentrations.

### Bacterial two-hybrid system and β-galactosidase activity.

A bacterial adenylate cyclase two-hybrid system (Euromedex, Souffelweyersheim, France) was used to validate interactions between DnaK, DksA, and DnaJ. The pKT25-cloned plasmids harboring DksA, DnaK, DnaJ, or RpoA as a N-terminal T25 fusion were cotransformed with the pUT18C vector encoding DksA, RpoA, or DnaJ variants as a C-terminal T18 fusion into E. coli BTH101. β-Galactosidase activity was determined in samples collected at stationary phase after 18 h of culture at 30°C in a shaker incubator. Enzyme activity expressed as Miller units was calculated as (*A*_420_ × 1,000)/[time (min) × *A*_600_ × cell volume (ml)] (27).

### Pulldown assays.

Interactions between recombinant proteins *in vitro* were analyzed using pulldown assays (27, 33). Reduced or oxidized GST-DksA proteins were prepared by treatment with 1 mM DTT or 100 μM H_2_O_2_ for 1 h at 37°C. One micromole of GST-DksA or GST-DnaK variant proteins (i.e., bait) was incubated for 2 h with 200 μl glutathione-Sepharose 4B beads (bioWORLD, Dublin, OH) in 50 mM Tris-HCl buffer, pH 7.5, at 4°C. The columns were washed with 20 bed volumes of 50 mM Tris-HCl buffer, pH 7.5, and incubated for 2 h at 4°C with rotation in the presence of 1 μmol of C-terminally 6×His-tagged wild-type DnaK, DnaJ, or DnaJ variants (i.e., prey). Where indicated, interactions of GST-DnaK variants with DnaJ-6×His variants were also tested. The columns were washed with 50 mM Tris-HCl buffer, pH 7.5, containing 50 mM NaCl. The proteins were eluted with 50 mM Tris-HCl buffer, pH 7.5, containing 500 mM NaCl. Eluted proteins were precipitated by 10% trichloroacetic acid (TCA). 6×His-tag fusion proteins were loaded onto 12% SDS-PAGE gels and detected by immunoblotting using a 1:1,000 dilution of a rabbit anti-6×His antibody (Rockland Immunochemicals, Limerick, PA), followed by a 1:10,000 dilution of goat anti-rabbit IgG conjugated with horseradish peroxidase (Pierce, Rockford, IL). The blots were processed using the ECL Prime Western blotting detection reagent (GE Healthcare Life Sciences) and visualized with a ChemiDoc XRS imaging system (Bio-Rad). Purified GST proteins were used as negative controls.

### Analysis of DksA redox by alkylation with AMS.

To detect thiol modification of recombinant DksA cysteine residues, 5 μM DksA was incubated for 1 h in 100 mM potassium phosphate buffer, pH 7.4, with 1 mM H_2_O_2_ at 37°C in the presence and absence of 5 μM DnaJ or DnaJ variants. Samples were precipitated by 15% TCA, resuspended in AMS buffer (1 M Tris-HCl [pH 8.0], 1 mM EDTA, 0.1% SDS, and 15 mM 4-acetamido-4′-maleimidyl stilbene-2,2′-disulfonic acid [AMS] [Thermo Fisher Scientific]), incubated at 37°C for 1 h in the dark, and loaded onto nonreducing 18% SDS-PAGE gels for visualization using Coomassie brilliant blue staining.

### Bacterial growth in response to heat.

Salmonella organisms grown overnight in LB broth at 30°C were diluted 1:100 in LB broth and cultured for 6 h at 30°C or 45°C. Bacterial growth was followed spectrophotometrically at *A*_600_.

### Motility assays.

Approximately 10^7^ Salmonella cells grown in LB broth overnight were spotted on the surfaces of 0.3% LB agar plates. The diameter of growth was measured in millimeters after 3.5 h of incubation at 37°C.

### Intracellular survival.

J774 cells were cultured in RPMI medium (Millipore-Sigma) supplemented with 10% heat-inactivated fetal bovine serum (Thermo Fisher Scientific), 1 mM sodium pyruvate (Thermo Fisher Scientific), 2 mM l-glutamine (Thermo Fisher Scientific) and 20 mM HEPES (Thermo Fisher Scientific). Confluent J774 cells were infected at a multiplicity of infection (MOI) of 2 with Salmonella grown overnight in LB broth at 30°C. Intracellular survival was assessed after cell host lysis by the addition of PBS containing 0.1% Triton X-100 (Thermo Fisher Scientific). Specimens were serially diluted in PBS, and the Salmonella burden was enumerated on LB agar plates after overnight growth. Fold replication was calculated from the number of bacteria recovered after 18 h of infection compared to that at time zero.

10.1128/mBio.03443-20.1 TEXT S1 Determination of zinc content. Download Text S1, DOC file, 0.06 MB. Copyright © 2021 Kim et al. 2021 Kim et al. https://creativecommons.org/licenses/by/4.0/ This content is distributed under the terms of the Creative Commons Attribution 4.0 International license.
