# Supplementary figures and images for: The DnaK/DnaJ Chaperone System Enables RNA Polymerase-DksA Complex Formation in Salmonella Experiencing Oxidative Stress
Source: mBio. 2021 May 11;12(3):e03443-20. doi: 10.1128/mBio.03443-20 (PMC8262869; doi:10.1128/mBio.03443-20)

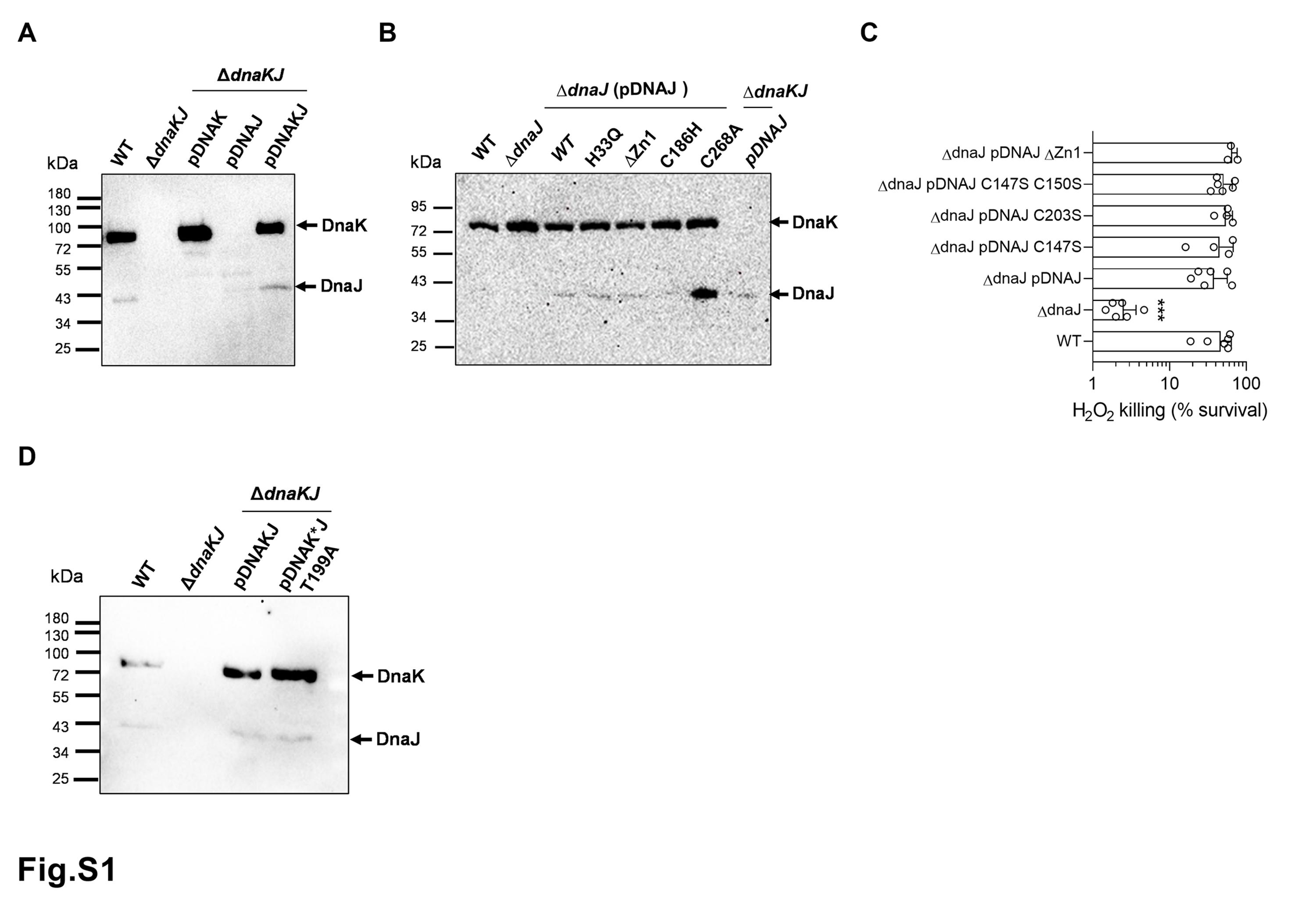

Supplement: FIG S1 [file mbio.03443-20-sf001.tif]

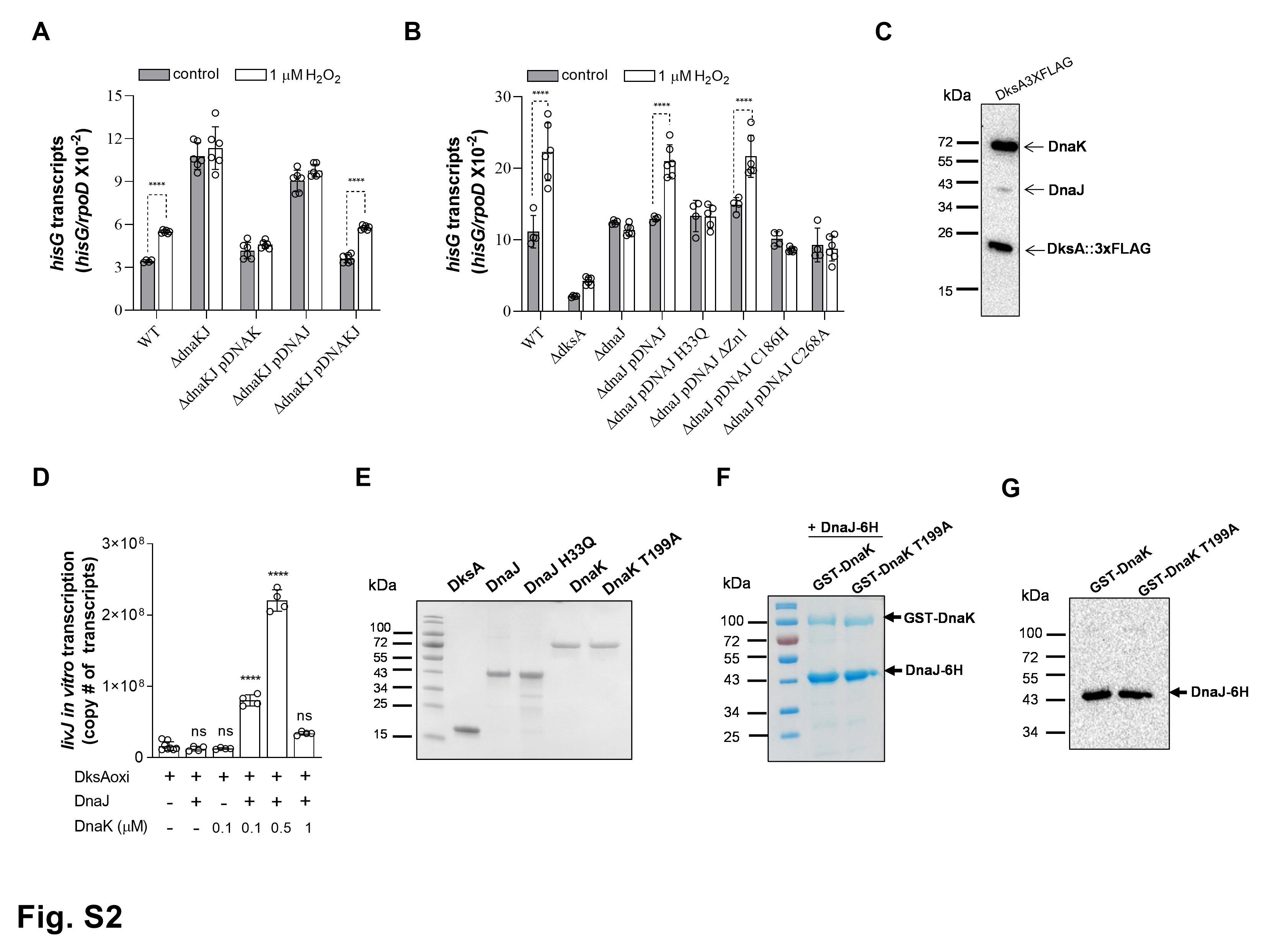

Supplement: FIG S2 [file mbio.03443-20-sf002.tif]

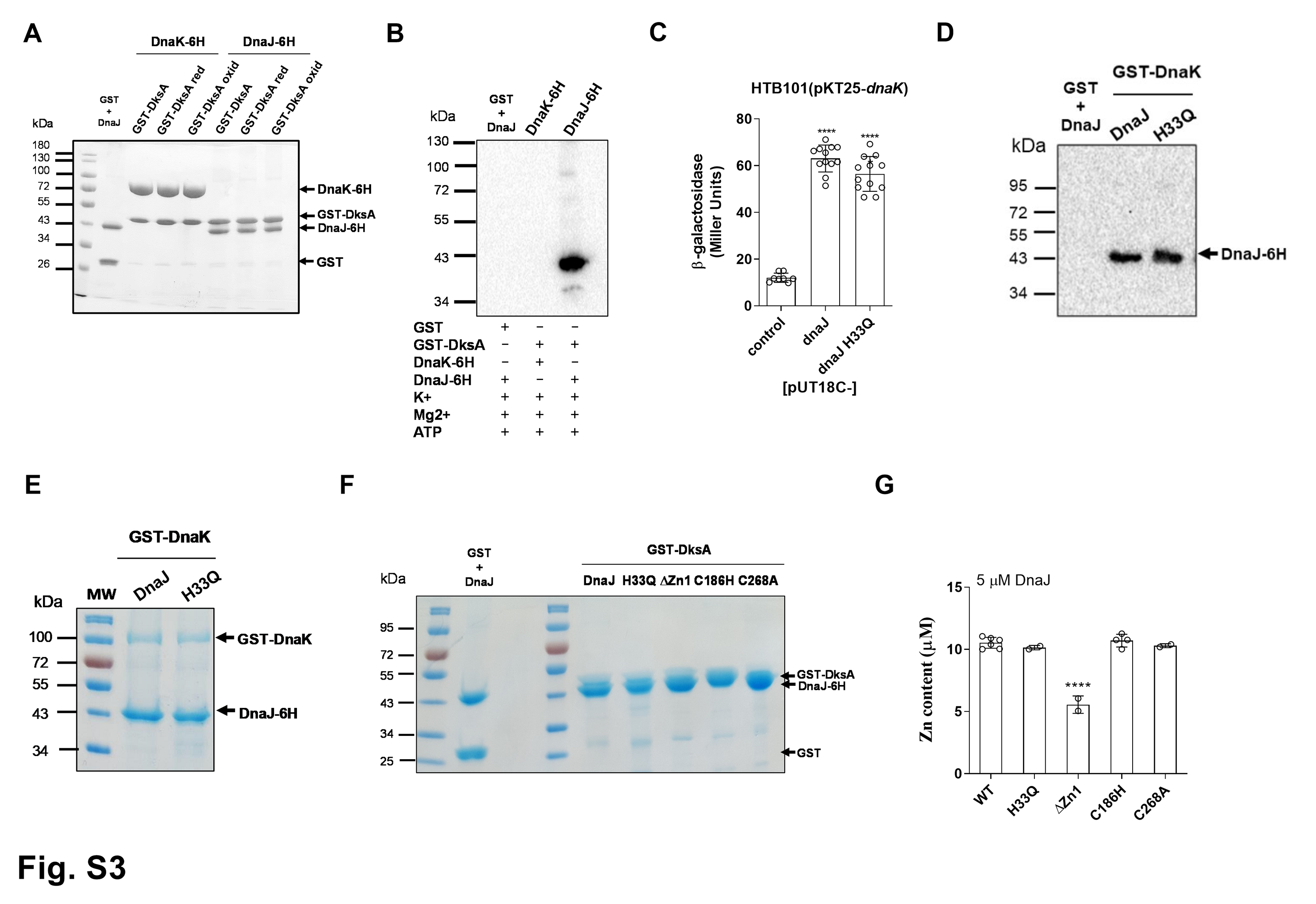

Supplement: FIG S3 [file mbio.03443-20-sf003.tif]

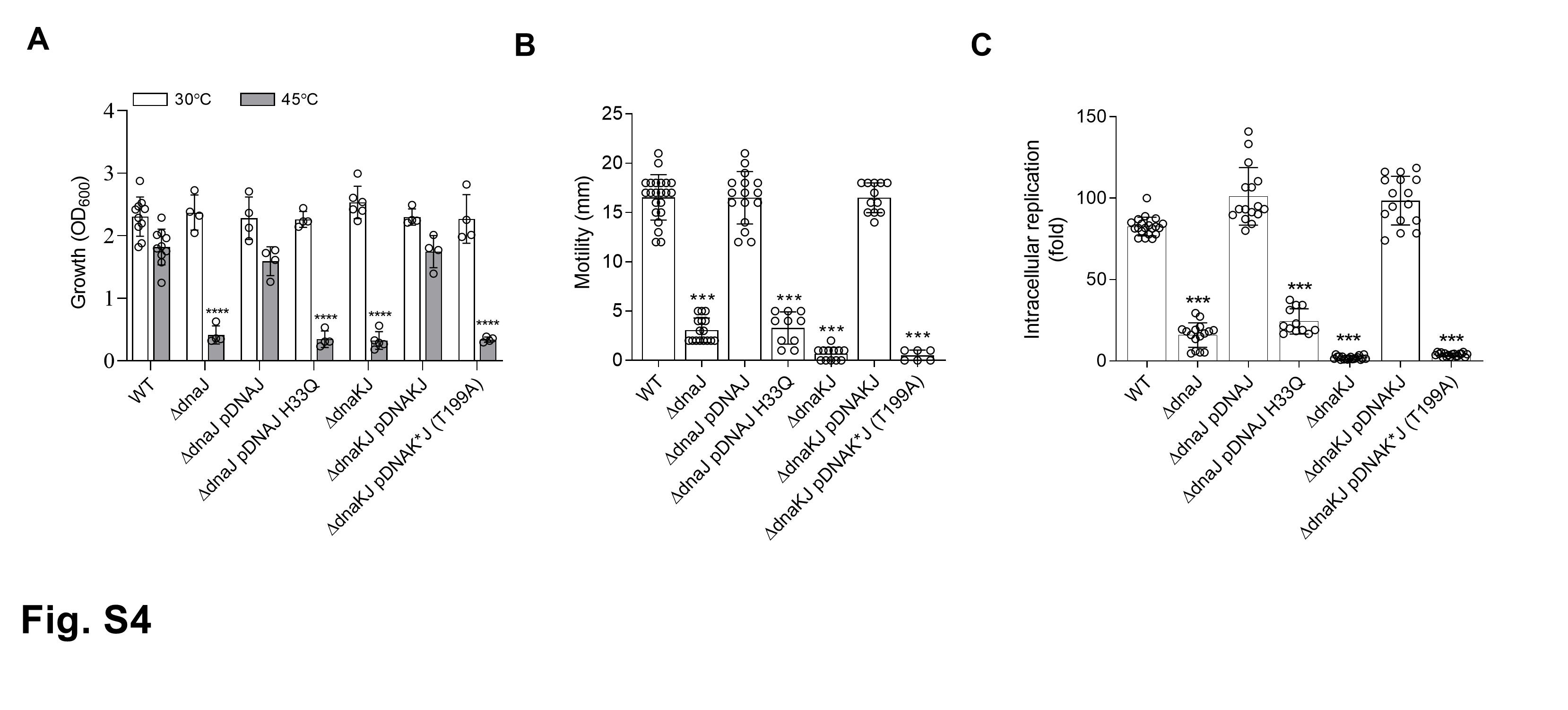

Supplement: FIG S4 [file mbio.03443-20-sf004.tif]
