# Supplementary material for: The DnaK/DnaJ Chaperone System Enables RNA Polymerase-DksA Complex Formation in Salmonella Experiencing Oxidative Stress
Source: mBio. 2021 May 11;12(3):e03443-20. doi: 10.1128/mBio.03443-20 (PMC8262869; doi:10.1128/mBio.03443-20)
Supplement: TABLE S1 [file mbio.03443-20-st001.doc]

**Table S1. Bacterial strains used in this study**.

Strain Relevant characteristics Reference

***Salmonella***

14028s wild-type of *S. Typhimurium* ATCC

AV06143 *dksA*::FRT lab strain

AV15172 Δ*dnaJ*::Km  2

AV15175 Δ*dnaKJ*::Km This study

AV15184 Δ*dnaJ*::Km (pWSK29::*dnaJ*) 2

AV18211 Δ*dnaJ*::Km (pWSK29::*dnaJ* H33Q) This study

AV18276 Δ*dnaJ*::Km (pWSK29::*dnaJ* ΔZn1) This study

AV16068 Δ*dnaJ*::Km (pWSK29::*dnaJ* C186H) 2

AV17055 Δ*dnaJ*::Km (pWSK29::*dnaJ* C268A) 2

AV17181 Δ*dnaJ*::Km (pWSK29::*dnaJ* ΔJ-GF) This study

AV19278 Δ*dnaKJ*::Km (pWSK29::*dnaK*) This study

AV18001 Δ*dnaKJ*::Km (pWSK29::*dnaJ*) This study

AV15182 Δ*dnaKJ*::Km (pWSK29::*dnaKJ*) This study

AV20028 Δ*dnaKJ*::Km (pWSK29::*dnaK** T199A *dnaJ*) This study

AV08016 *dksA*::3XFLAG 2

*E.coli*

DH5α *supE*44 Δ*lacU*169 (φ80 *lacZ* ΔM15) *hsdR*17 *recA*1 3  *endA*1 *gyrA*96 *thi-*1 *relA*1

BTH101 F-, *cya*-99, *ara*D139, *gal*E15, *gal*K16, *rps*L1 (Str^r^), Euromedex

*hsd*R2, *mcr*A1, *mcr*B1

BL21(DE3) fhuA2 [lon] ompT gal (λ DE3) [dcm] ∆hsdS Invitrogen

λ DE3 = λ sBamHIo ∆EcoRI-B

int::(lacI::PlacUV5::T7 gene1 i21 ∆nin5

AV19126 BTH101 (pKT25::*dnaK,* pUT18C) This study

AV19127 BTH101 (pKT25::*dnaK,* pUT18C::*dnaJ*) This study

AV19139 BTH101 (pKT25::*dnaK,* pUT18C::*dnaJ* H33Q) This study

AV20034 BTH101 (pKT25::*dnaK,* pUT18C::*dksA*) This study

AV20033 BTH101 (pKT25::*rpoA*, pUT18C::*dksA*) This study

AV20090 BTH101 (pKT25::*dnaJ*, pUT18C::*dksA*) This study

AV20031 BTH101 (pKT25, pUT18C::*dksA*) This study

AV15092 BTH101 (pKT25::*dksA,* pUT18C) 2

AV15098 BTH101 (pKT25::*dksA,* pUT18C::*rpoA*) 2

AV15096 BTH101 (pKT25::*dksA,* pUT18C::*dnaJ*) 2

AV19138 BTH101 (pKT25::*dksA,* pUT18C::*dnaJ* H33Q) This study

AV20147 BTH101 (pKT25::*dksA,* pUT18C::*dnaJ* ΔZn1) This study

AV20148 BTH101 (pKT25::*dksA,* pUT18C::*dnaJ* C186H) This study

AV20149 BTH101 (pKT25::*dksA,* pUT18C::*dnaJ* C268A) This study

AV10267 BL21(DE3) (pGEX6p-DksA) 4

AV19042 BL21(DE3) (pGEX6p-DnaK) This study

AV19076 BL21(DE3) (pGEX6p-DnaK T199A) This study

AV17138 BL21(DE3) (pET22b-DnaJ) 2

AV20028 BL21(DE3) (pET22b-DnaJ H33Q) This study

AV20029 BL21(DE3) (pET22b-DnaJ ΔZn1) This study

AV17139 BL21(DE3) (pET22b-DnaJ C186H) 2

AV17140 BL21(DE3) (pET22b-DnaJ C268A) 2

AV18263 BL21(DE3) (pET22b-DnaK) This study
