# Supplementary material for: The DnaK/DnaJ Chaperone System Enables RNA Polymerase-DksA Complex Formation in Salmonella Experiencing Oxidative Stress
Source: mBio. 2021 May 11;12(3):e03443-20. doi: 10.1128/mBio.03443-20 (PMC8262869; doi:10.1128/mBio.03443-20)
Supplement: TABLE S2 [file mbio.03443-20-st002.doc]

**Table S2. Plasmids used in this study**.

Plasmid Relevant characteristics Reference

pET22b(+) *ori* pBR322, C-terminal His·Taq fusion vector, Pn^r^ Novagen

pET22b::DnaJ pET-22b(+) + 1.1-kb DNA containing *dnaJ*, Pn^r^ 2

pET22b::DnaJ H33Q pET-22b(+) + 1.1-kb DNA containing *dnaJ* H33Q, Pn^r^ This study

pET22b::DnaJ ΔZn1 pET-22b(+) + 1.1-kb DNA containing *dnaJ* ΔZn1, Pn^r^ This study

pET22b::DnaJ C186H pET-22b(+) + 1.1-kb DNA containing *dnaJ* C186H, Pn^r^ 2

pET22b::DnaJ C268A pET-22b(+) + 1.1-kb DNA containing *dnaJ* C268A, Pn^r^ 2

pET22b::DnaK pET-22b(+) + 1.94-kb DNA containing *dnaK*, Pn^r^ This study

pGEX6p::DksA pGEX6p + 454-bp DNA containing *dksA*, Pn^r^ 4

pGEX6p::DnaK pGEX6p + 1.94-kb DNA containing *dnaK*, Pn^r^ This study

pGEX6p::DnaK T199A pGEX6p + 1.94-kb DNA containing *dnaK* T199A, Pn^r^ This study

pKD13 template vector for FRT-flanked Km^r^ cassette, Km^r^ Pn^r^ 5

pKT25 pSU40 derivative with T25 domain of CyaA, Euromedex

MCS at the end of T25, Km^r^

pKT25::*dksA* pKT25 plasmid with *cyaAT25-dksA* fusion, Km^r^ 2

pKT25::*dnaJ* pKT25 plasmid with *cyaAT25-dnaJ* fusion, Km^r^ This study

pKT25::*rpoA* pKT25 plasmid with *cyaAT25-rpoA* fusion, Km^r^ 2

pKT25::*dnaK* pKT25 plasmid with *cyaAT25-dnaK* fusion, Km^r^ This study

pTIM in vitro transcription backbone plasmid, 6

bla *rrnB* & *rpoC* term pBluescript, Pn^r^

pTIM::*livJ* pTim *+* 1.34-kb DNA containing P*livJ* (-240) and *livJ* 2

pTIM::*hisG* pTim *+* 0.80-kb DNA containing P*hisG*(-351) and *hisG*  2

pUT18C pUC19 derivative with T18 domain of CyaA, Euromedex

MCS at the 3’ start of T18, Pn^r^

pUT18C::*dnaJ* pUT18 plasmid with *dnaJ-cyaAT18* fusion, Pn^r^ 2

pUT18C::*dnaJ* H33Q pUT18 plasmid with *dnaJ* H33Q*-cyaAT18* fusion, Pn^r^ This study

pUT18C::*dnaJ* ΔZn1 pUT18 plasmid with *dnaJ* ΔZn1*-cyaAT18* fusion, Pn^r^ This study

pUT18C::*dnaJ* C186H pUT18 plasmid with *dnaJ* C186H*-cyaAT18* fusion, Pn^r^ This study

pUT18C::*dnaJ* C268A pUT18 plasmid with *dnaJ* C268A*-cyaAT18* fusion, Pn^r^ This study

pUT18C::*dksA* pUT18 plasmid with *dksA-cyaAT18* fusion, Pn^r^ This study

pWSK29 low copy plasmid, *lacZα*, Pn^r^  7

pDNAJ pWSK29 + 1.49-kb DNA containing p*dnaK::dnaJ*, Pn^r^ 2

pDNAJ H33Q pWSK29 + 1.49-kb DNA containing p*dnaK::dnaJ* H33Q, Pn^r^ This study

pDNAJ ΔZn1 pWSK29 + 1.49-kb DNA containing p*dnaK::dnaJ* ΔZn1, Pn^r^ This study

pDNAJ C186H pWSK29 + 1.49-kb DNA containing p*dnaK::dnaJ* C186H, Pn^r^ 2

pDNAJ C268A pWSK29 + 1.49-kb DNA containing p*dnaK::dnaJ* C268A, Pn^r^ 2

pDNAJ ΔJ-GF pWSK29 + 1.09-kb DNA containing p*dnaK::dnaJ* ΔJ-GF, Pn^r^ This study

pDNAK pWSK29 + 2.28-kb DNA containing p*dnaK::dnaK*, Pn^r^ This study

pDNAKJ pWSK29 + 3.54-kb DNA containing p*dnaK::dnaKJ*, Pn^r^ This study

pDNAK*J T199A pWSK29 + 3.54-kb DNA containing p*dnaK::dnaK* T199A This study

& *dnaJ*, Pn^r^
