# Supplementary material for: The DnaK/DnaJ Chaperone System Enables RNA Polymerase-DksA Complex Formation in Salmonella Experiencing Oxidative Stress
Source: mBio. 2021 May 11;12(3):e03443-20. doi: 10.1128/mBio.03443-20 (PMC8262869; doi:10.1128/mBio.03443-20)
Supplement: TABLE S3 [file mbio.03443-20-st003.doc]

**Table S3. Oligonucleotides used in this study.**

| **Strains** | **Primer Sequence (5’ → 3’)** |
| --- | --- |
| Δ*dnaKJ*::Km | F: ACCACATGATGACCGATTTTTAGTGGAGACGTTTAGATGGT GTAGGCTGGAGCTGCTTC |
|  | R: ACACCCGGGCTGAAGAAAAATACAACGGGAAAAGATTAATT  CCGGGGATCCGTCGACC |
|  |  |
| **Plasmid** |  |
| pET22b::DnaK  pGEX6p::DnaK | F : ATCGCATATGGGTAAAATTATTGGTATCG |
|  | R : ATCGCTCGAGTTTTTTATCTTTTACTTCTTC  F : ATCGGGATCCGGTAAAATTATTGGTATCGA  R : ATCGCTCGAGTTATTTTTTATCTTTTACTTC |
| pKT25::*dnaJ* | F : ATCGTCTAGAGGCGAAAAGAGATTACTACGA |
|  | R : ATCAGGATCCCTTAGCGAGTCAAATCGTCAA |
| pKT25::*dnaK* | F : ATCGTCTAGAGGGTAAAATTATTGGTATC  R : ATCGGGATCCTCTTTTTTATCTTTTACTTC |
| pUT18C::*dksA* | F : TCTAGAGCAAGAAGGGCAAAACCGTAAAAC |
|  | R : GGATCCCCCGCCATCTGTTTTTCGCG |
| pDNAJ ΔJ-GF  1.*pdnaK* DNA |  |
|  | F : ATTGGGTACCGGGCCCCCCCTCGAGCTCATGGCAACGATT  CAG (Gibson)  R : TATAACGCAA**CATCTAAACG**TCTCCACTAAAAATTC (Gibson) |
| 2. *dnaJ* ΔJ-GF DNA | F : **CGTTTAGATG**TTGCGTTATAACATGGATC (Gibson)  R : CCACCGCGGTGGCGGCCGCTCTAGATTAGCGAGTCAAATC  GTC (Gibson) |
| pDNAK | F : ATCGCTCGAGAGCAATACCGTCTATCATACTCTG |
|  | R : ATCGTCTAGAGAAACCTCTTCGCCCGTGCC |
| pDNAKJ | F : CCGCTCGAGGCAGGCCGACGGAAATCGTTAACAC |
|  | R: CGCGGATCCTTAGCGAGTCAAATCGTCAAAGAATTTTTTCA  CG |
|  |  |
| **Point mutation** | |
| *dnaJ* H33Q | F: CTGGCCATGAAATAT*CAA*CCGGACCGCAATCAG |
|  | R: CTGATTGCGGTCCGG*TTG*ATATTTCATGGCCAG |
| *dnaJ* ΔZn1 set 1 | F: CGCTGGAGGAG*AGC*GACGTT*AGC*CACGGCAGCGGC  R: GCCGCTGCCGTG*GCT*AACGTC*GCT*CTCCTCCAGCG |
| set 2 | F: CAAAGATCCG*AGT*CATAAA*AGT*CACGGTCATGGGC  R: GCCCATGACCGTG*ACT*TTTATG*ACT*CGGATCTTTG |
| *dnaJ* C186H | F: CCCTGACAGTGTGGG*TGG*GTCTGCTGTACAGC  R: GCTGTACAGCAGAC*CCA*CCCACACTGTCAGGG |
| *dnaJ* C268A | F: CAATAATCTTTAT*GCA*GAAGTGCCGATCAACTTTG  R: GATCGGCACTTC*TGC*ATAAAGATTATTGCC |
| *dnaK* T199A | F: GACCTCGGTGGTGGT*GCT*TTCGATATCTCTATT |
|  | R: AATAGAGATATCGAA*AGC*ACCACCACCGAGGTC |
|  |  |
| **Real time qRT-PCR** | |
| *rpoD* | F: gtggcttgcaattccttgat |
|  | R: agcatctggcgagaaata |
|  | Probe: 6-FAM-ataagttcgaataccgtcgcg-3BHQ-1 |
| *livJ* | F: cgcagggctgaaaaccca |
|  | R: cacacgaatgcgccgcta |
|  | Probe: 6-FAM-tcagcggaaggcttactggtc-3BHQ-1 |
| *hisG* | F : CAGGCCGTTTAAGCGATGATTCACGAG |
|  | R : AATACCGAGATCGACCACGCCATCC |
|  | Probe: 6-FAM-ATCGGCATGTTTTCCGCCATCGCAATCAGG-  3BHQ-1 |
|  |  |
|  |  |

* Restriction enzyme sites are underlined.

** Point mutation sites are indicated in italic.

*** Primers designed by NEBuilder Assembly Tool are presented as (Gibson). Overlap nucleotide sequence for Gibson cloning are indicated in bold.
