# Supplementary material for: The DnaK/DnaJ Chaperone System Enables RNA Polymerase-DksA Complex Formation in Salmonella Experiencing Oxidative Stress
Source: mBio. 2021 May 11;12(3):e03443-20. doi: 10.1128/mBio.03443-20 (PMC8262869; doi:10.1128/mBio.03443-20)
Supplement: TEXT S1 [file mbio.03443-20-s0001.doc]

**SUPPLEMENTAL MATERIAL**

The DnaK/DnaJ chaperone system enables RNA polymerase-DksA complex formation in *Salmonella* experiencing oxidative stress

Ju-Sim Kim, Lin Liu, and Andrés Vázquez-Torres

**SUPPLEMENTAL MATERIALS AND METHODS**

**Determination of zinc content.** 5 µM of DnaJ variants were treated with 150 PAR [(4-(2-Pyridylaso) resorcinol] (Sigma, St. Louis, MO) at 37 °C for 1 h in the presence of 8 M urea. The zinc-PAR chelate was quantified spectrometrically at OD_500_. Zinc concentrations were calculated from a standard curve with ZnCl_2_ (1).

**SUPPLEMENTAL REFERENCES**

1. Crawford M A, Tapscott T, Fitzsimmons L F, Liu L, Reyes A M, Libby S J, Trujillo M, Fang F C, Radi R, and Vazquez-Torres A. 2016. Redox-active sensing by bacterial DksA transcription factors is determined by cysteine and zinc content. mBio 7: e02161-02115.

2. Kim J S, Liu L, Fitzsimmons L F, Wang Y, Crawford M A, Mastrogiovanni M, Trujillo M, Till J K A, Radi R, Dai S, and Vazquez-Torres A. 2018. DksA-DnaJ redox interactions provide a signal for the activation of bacterial RNA polymerase. Proc Natl Acad Sci U S A 115:E11780-E11789.

3. Hanahan D. 1983. Studies on transformation of *Escherichia coli* with plasmids. J Mol Biol 166:557-580.

4. Henard C A, Tapscott T, Crawford M A, Husain M, Doulias P T, Porwollik S, Liu L, McClelland M, Ischiropoulos H, and Vazquez-Torres A. 2014. The 4-cysteine zinc-finger motif of the RNA polymerase regulator DksA serves as a thiol switch for sensing oxidative and nitrosative stress. Mol Microbiol 91:790-804.

5. Datsenko K A, and Wanner B L. 2000. One-step inactivation of chromosomal genes in *Escherichia coli* K-12 using PCR products. Proc Natl Acad Sci U S A 97:6640-6645.

6. Tapscott T, Kim J S, Crawford M A, Fitzsimmons L, Liu L, Jones-Carson J, and Vazquez-Torres A. 2018. Guanosine tetraphosphate relieves the negative regulation of *Salmonella* pathogenicity island-2 gene transcription exerted by the AT-rich *ssrA* discriminator region. Sci Rep 8:9465.

7. Wang R F, and Kushner S R. 1991. Construction of versatile low-copy-number vectors for cloning, sequencing and gene expression in *Escherichia coli.* Gene 100: 195-199.

**SUPPLEMENTAL FIGURE LEGENDS:**

**Figure S1. Determination of DnaK and DnaJ expression of *Salmonella* strains.** (A, B and D) Expression of DnaK and/or DnaJ proteins were measured in Immunoblot assays in the indicated *Salmonella* strains grown to exponential phase (OD_600_ of 0.7 ~ 0.8) in LB broth. 30 µg of lysates were loaded onto 10% SDS-PAGE gels. The blots were probed with a 1:500 of anti-DnaK mouse antibody (MBL, Woburn, MA) or a 1:1,000 of anti-DnaJ rabbit antibody (Enzo, Farmingdale, NY), followed by a 1:10,000 dilution of goat anti-mouse IgG (Pierce, Rockford, IL) or goat anti-rabbit IgG (Pierce, Rockford, IL) conjugated with horseradish peroxidase (HRP). The blot is representative of 2-3 independent experiments. (C) Survival of wild-type and the indicated *Salmonella* mutants grown overnight in LB broth after 2 h of treatment with 200 µM H_2_O_2_ in PBS. The data are the mean ± SD (n = 4-6) from 2-4 individual experiments. ***, *p* < 0.001 as determined by one-way ANOVA.

**Figure S2. Transcription of *hisG* in *Salmonella* strains and titration of DnaK concentration of *in vitro* transcription.** (A and B) Abundance of *hisG* transcripts in the indicated *Salmonella* strains grown anaerobically in EGCA medium. Where indicated, some of the samples were treated with or without 1 μM H_2_O_2_ for 30 min. The abundance of *hisG* transcripts was normalized to the amount of *rpoD* mRNA within samples. ****, *p*<0.0001 as determined by one-way ANOVA. Data are the mean ± SD (n= 4-6) from 2-3 independent experiments. (C) Expression of DnaK, DnaJ and DksA3XFLAG proteins were measured by Western blotting in specimens isolated from a *dksA*::3XFLAG *Salmonella* strain grown to exponential phase (OD_600_ of 0.6) in LB broth. 50 µg of lysates were loaded onto AnyKD SDS-PAGE gels (Bio-Rad). The blots were probed with a 1:500 of anti-DnaK mouse antibody (MBL, Woburn, MA), a 1:1,000 of anti-DnaJ rabbit antibody (Enzo, Farmingdale, NY) and a 1:500 of anti-FLAG mouse antibody (Millipore Sigma, St. Louis, MO), followed by a 1:10,000 dilution of goat anti-mouse IgG (Pierce, Rockford, IL) and goat anti-rabbit IgG (Pierce, Rockford, IL) conjugated with horseradish peroxidase (HRP). The blot is representative of 4-5 independent experiments. (D) Effect of increasing DnaK concentrations in *livJ* *in vitro* transcription. Reactions contained 5 µM of oxidized DksA and 50 nM of DnaJ. *livJ in vitro* transcription was determined by real-time qRT-PCR. The data are the mean ± SD (n = 4-8) from at least 2 individual experiments. ****, *p* < 0.0001 as determined by one-way ANOVA. ns, nonsignificant compared to reactions containing only 5 µM of oxidized DksA. (E) Purified *Salmonella* untagged DksA, DnaK and DnaK T199A proteins and DnaJ-6H and DnaJ H33Q-6H proteins used in *in vitro* transcription assays were assessed by AnykD SDS-PAGE (Bio-Rad) and Coomassie Brilliant Blue staining. (F) Input proteins used in the biochemical pull-down assays in Fig. S2G were evaluated by 10% SDS PAGE gels and Coomassie Brilliant Blue staining. (G) DnaJ proteins in the biochemical pull-down assays using recombinant GST-DnaK or GST-DnaK T199A proteins were detected by Immunoblot. Data are representative from 2 independent experiments.

**Figure S3. Characterization of DnaK binding to DnaJ H33Q and determination of zinc content of recombinant DnaJ variants.** (A, E and F) Input proteins used in the biochemical pull-down assays in Fig. 6B, S3D and 6C were evaluated by 10% SDS PAGE gels and Coomassie Brilliant Blue staining. (B) The binding of GST-DksA to DnaK-6H or DnaJ-6H C-terminal tagged proteins was assessed using a biochemical pull-down assay in the presence of 2 mM MgCl_2_, 60 mM potassium glutamate and 200 μM ATP by Immunoblot. Data are representative of 2 independent experiments. (C) DnaK and DnaJ H33Q interactions were evaluated in a bacterial two-hybrid system in which T25 and T18 fragments of adenylate cyclase fused to DnaK and DnaJ H33Q proteins. DnaJ was used for comparison. The data are the mean ± SD (n = 6-12) from 3-4 individual experiments. ****, *p* < 0.0001 as determined by one-way ANOVA. (D) Biochemical pull-down assays using recombinant DnaK and DnaJ H33Q proteins was performed to confirm their binding. GST and GST-DnaK proteins were used as bait, whereas DnaJ-6H and DnaJ H33Q-6H proteins were used as prey. DnaJ variants in the pull-down assays were detected by Immunoblot. Data are representative from 2-3 independent experiments. (G) Determination of zinc content from 5 μM of recombinant DnaJ-6H variants used in the biochemical pull-down and AMS assays was measured with the zinc chelator PAR (1). The data are the mean ± SD (n = 2-6) from at least 2 individual experiments. ****, *p* < 0.0001 as determined by one-way ANOVA.

**Figure S4. Effect of DnaJ H33Q and DnaK T199A mutations on thermotolerance, motility and pathogenesis of *Salmonella*.** (A) Growth of the indicated *Salmonella* stains in LB broth for 6 h at 30ºC or 45ºC. The data are the mean ± SD (n = 4-10) from 3-6 individual experiments. ****, *p* < 0.0001 as determined by one-way ANOVA. (B) Swimming capacity of the indicated *Salmonella* strains grown in LB broth overnight. 10^7^ CFU were spotted onto 0.3% LB agar plates and incubated for 3.5 h. The data are the mean ± SD (n = 6-22) from at least 4 individual experiments. ***, *p* < 0.001 as determined by one-way ANOVA. (C) Intracellular replication of the indicated *Salmonella* strains in J774 cells at 18 h post-infection was determined by CFU measurement. The data are the mean ± SD (n = 12-20) from at least 3 independent experiments. ***, *p* < 0.001 as determined by one-way ANOVA.
